# Antigenic evolution of viruses in host populations

**DOI:** 10.1371/journal.ppat.1007291

**Published:** 2018-09-12

**Authors:** Igor M. Rouzine, Ganna Rozhnova

**Affiliations:** 1 Sorbonne Université, Institute de Biologie Paris-Seine, Laboratoire de Biologie Computationelle et Quantitative, LCQB, F-75004 Paris, France; 2 Institute of Theoretical Physics, University of Cologne, Germany; 3 BioISI – Biosystems and Integrative Sciences Institute, Faculdade de Ciências, Universidade de Lisboa, Lisboa, Portugal; 4 Julius Center for Health Sciences and Primary Care, University Medical Center Utrecht, Utrecht, The Netherlands; Institut Pasteur, FRANCE

## Abstract

To escape immune recognition in previously infected hosts, viruses evolve genetically in immunologically important regions. The host’s immune system responds by generating new memory cells recognizing the mutated viral strains. Despite recent advances in data collection and analysis, it remains conceptually unclear how epidemiology, immune response, and evolutionary factors interact to produce the observed speed of evolution and the incidence of infection. Here we establish a general and simple relationship between long-term cross-immunity, genetic diversity, speed of evolution, and incidence. We develop an analytic method fusing the standard epidemiological susceptible-infected-recovered approach and the modern virus evolution theory. The model includes the factors of strain selection due to immune memory cells, random genetic drift, and clonal interference effects. We predict that the distribution of recovered individuals in memory serotypes creates a moving fitness landscape for the circulating strains which drives antigenic escape. The fitness slope (effective selection coefficient) is proportional to the reproductive number in the absence of immunity *R*_0_ and inversely proportional to the cross-immunity distance *a*, defined as the genetic distance of a virus strain from a previously infecting strain conferring 50% decrease in infection probability. Analysis predicts that the evolution rate increases linearly with the fitness slope and logarithmically with the genomic mutation rate and the host population size. Fitting our analytic model to data obtained for influenza A H3N2 and H1N1, we predict the annual infection incidence within a previously estimated range, (4-7)%, and the antigenic mutation rate of *U*_*b*_ = (5 − 8) ⋅ 10^−4^ per transmission event per genome. Our prediction of the cross-immunity distance of *a* = (14 − 15) aminoacid substitutions agrees with independent data for equine influenza.

## Author summary

Spread of many RNA viruses in a population represents a competition between host immune responses and viral evolution. RNA viruses accumulate mutations in immunologically important regions to escape immune recognition in hosts previously exposed to infection, while the immune system responds by producing new memory cells. Despite recent advances in data collection and their analysis, it remains conceptually unclear how epidemiology, immune response, and evolutionary factors interact to produce the observed speed of evolution and its incidence. By combining the standard epidemiological approach with the modern theory of viral evolution, we predict a general relationship between long-term cross-immunity, antigenic diversity of virus, its evolution speed, infection incidence, and the time to the most recent common ancestor. We apply these theoretical findings to available data on influenza virus to determine two important parameters of its evolution and confirm the model. Current strategies of vaccination against influenza should take into account stochastic fluctuations in fitness effect of mutations predicted by the theory.

## Introduction

Spread of many RNA viruses occurs as a race between host immune responses and rapid viral evolution. The development of treatment and effective preventive measures such as vaccines and therapeutic interference particles [1–3] requires understanding of the mechanics of viral evolution at the scale of a population. To evade immune recognition by hosts previously exposed to infection, in a never-ending chase, viruses accumulate mutations in immunologically relevant regions of the genome [4]. Despite advances in the collection and analysis of epidemiological and genomic data, it remains conceptually unclear how epidemiology, immune response, and evolutionary factors interact to produce the observed evolution speed and the incidence of infection.

Influenza virus infects 5-15% of the world population. The global spread and reinfection of the same individuals is caused by rapid evolution of antibody-binding regions [4]. A large amount of information has been obtained, including world-wide circulation [5–7], genetic maps of virus variants and antibodies, molecular mechanisms, and fitness effect of specific mutations [4, 8–10]. Vigorous data analysis and computer simulation helped to understand many features of influenza virus evolution [7, 11–15]. In particular, recent work [15] offers an inference model to predict short-term evolution of influenza, which is helpful for optimization of vaccination strategy. However, the more general connection between the population-scale viral parameters and its evolutionary behavior is still lacking.

The aim of this work is to establish general and simple relationships for the speed of virus evolution, genetic diversity, and annual incidence in terms of population parameters, and to train them on the available data for influenza virus. We propose a general analytic approach combining a susceptible-infected-recovered (SIR) framework [11, 16] with the stochastic evolution theory [17–25]. Using the experimental observation that phylogenetic trees of influenza virus have a vine-like structure with short branches [4], we focus on virus evolution along the one-dimensional trunk. Analysis demonstrates that the evolution under immune memory occurs in the form of a traveling wave in antigenic space, with fitness landscape moving together with the wave. The fitness slope (effective selection coefficient) can be expressed in terms of the cross-immunity distance.

We provide analytic predictions for the speed, incidence, and the average time to most recent common ancestor in terms of population parameters, including reproduction number, population size, and cross-immunity distance. Then we discuss how the punctuated nature of influenza evolution alternating small-effect and large mutations [4, 14] may be interpreted from the stochasticity of evolution.

## Model and methods

### Strain-structured epidemiological model

We start by describing briefly our model and approach. The details are given in S1 Appendix. Standard models of evolution focus on the dynamics of virus strains (variants), while standard epidemiological models study the transmission of a virus in a host population. For viruses that evolve to evade immune memory of previously infected hosts, evolutionary and epidemiological dynamics are tightly coupled [26]. Here we adopt a strain-based formulation of epidemiological models, in which all individuals are infected or recovered. Recovered individuals are classified according to their current ability to respond to various viral strains which represent genetic variants of an antibody-binding region of the virus (e.g., hemaglutinin gene for influenza virus). Each infected individual is assumed to be infected with a single strain denoted by *x*. We measure the “antigenic coordinate” *x* as genetic distance in terms of non-synonymous nucleotide substitutions. Infection by a viral strain is cleared in several days or weeks leaving in the recovered individual immunological memory that provides full protection against the same strain and partial protection against infection by genetically close strains. We assume one-dimensional space, *x*, that represents the trunk of the phylogenetic tree. For each recovered individual, we keep track only of the memory of the most recent infection [11, 12]. In S1 Appendix, Section 1.3.3, we show that this approximation has a modest effect on the final results.

Let *i*(*x*, *t*) denote the density of individuals currently infected with strain *x*, and *r*(*x*, *t*) be the density of individuals whose last infection was with strain *x* and who then recovered. The model is represented by a system of differential equations that describe the dynamics of the distributions *i*(*x*, *t*) and *r*(*x*, *t*):

$$\begin{array}{c} \frac{dr(x,t)}{dt}=-r\left(x,t\right)R_{0}\int_{x}^{\infty }K\left(x-y\right)i\left(y,t\right)d\;y+i\left(x,t\right),\\ \frac{di(x,t)}{dt}=i\left(x,t\right)[R_{0}\int_{-\infty }^{x}K\left(y-x\right)r\left(y,t\right)d\;y-1]\\ +(\text{mutation}\;\text{term}) \end{array}$$ (1)

We assume that each individual is either infected or recovered, as given by the normalization condition

$$\begin{array}{c} \int_{-\infty }^{+\infty }[r(x,t)+i(x,t)]\text{d}\;x=1. \end{array}$$

The treatment of mutations, which are assumed to be rare, will be described below in subsection *Mutation*.

Eq 1 describe the following epidemiological processes. Firstly, recovered individuals from strain *x* can be infected with strain *y* and their susceptibility is proportional to the cross-immunity function *K*(*x* − *y*), which depends on the genetic distance between strains *x* and *y*, so that *K*(*x* − *y*) > 0, *y* > *x*; *K*(*x* − *y*) ≡ 0, *y* < *x*; *K*(−∞) = 1.

**Fig 1:**
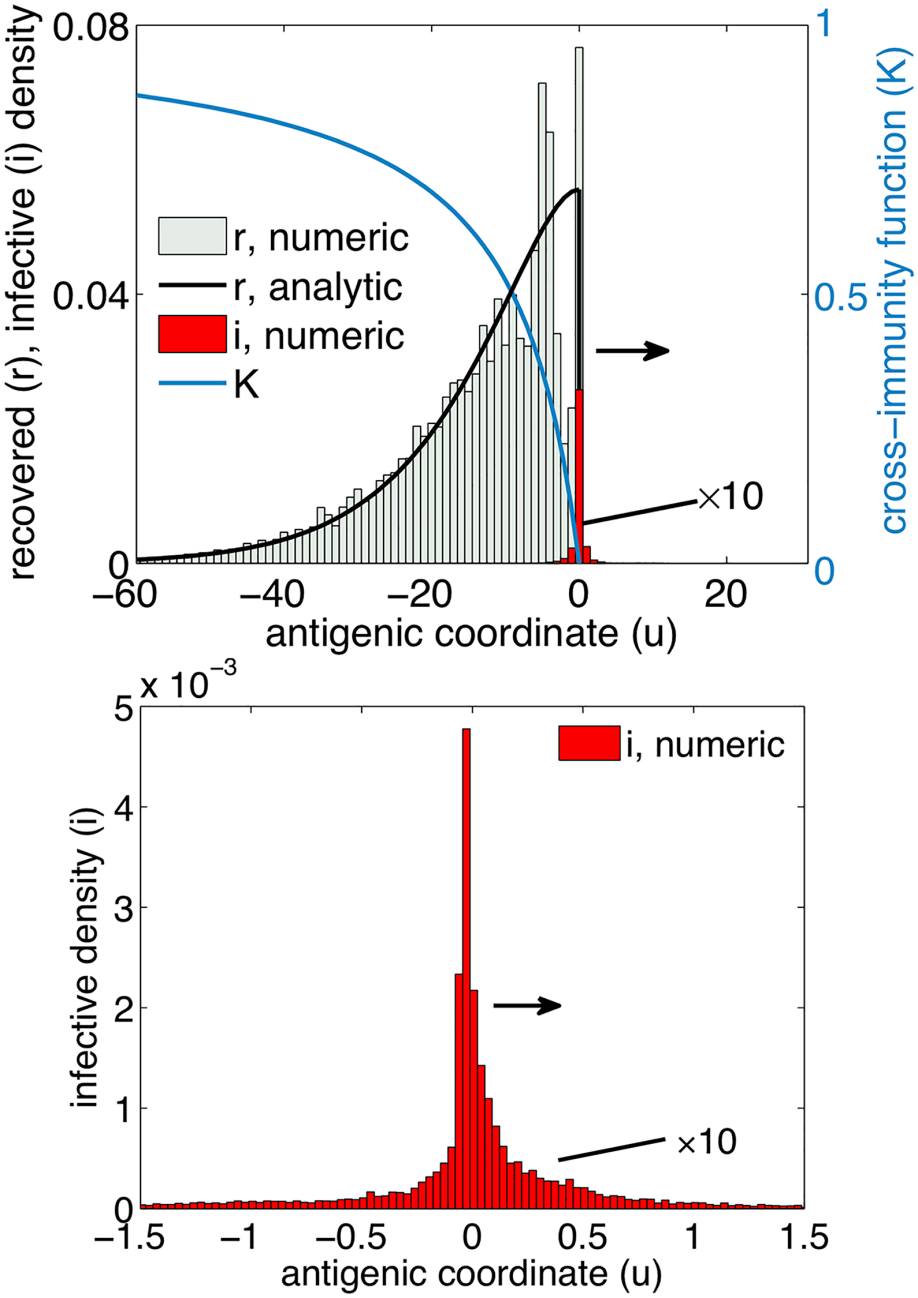
One-dimensional epidemiological model predicts a steady traveling wave along fitness axis. A) Frequencies of recovered individuals (black curve) and the infected individuals (red histogram) in population in the reference frame moving with the wave. Here X axis plots the antigenic coordinate in that reference frame, *u* = *x* − *ct*. Black solid line shows analytic prediction for *r*(*u*) (Eq 3). Histograms show the result of a full stochastic simulation of the epidemiological model, Eq 1. Blue line is cross-immunity function *K*(*u*) (Table 1). Parameters (example): *R*_0_ = 2, *a* = 9, *U*_*b*_ = 5.8 × 10^−6^, *N* = 10^8^. Units of the values on the axes are given in Table 1 and Eq 1. A wave in the rest frame of reference is shown in S2B Fig.

Here we assume that individuals recovered from strain *x* can be infected only by strains *y* ahead of *x* in time, *y* > *x*, so that *K*(*u*) is zero when its argument *u* is zero or positive (Fig 1, blue curve). In fact, there is a narrow region at the leading edge, where the backward infection could be possible. However, since the edge region is very narrow in the parameter range of interest, this process has a minor effect on the results (see the details in S1 Appendix, Section 1.3.2).

Secondly, infected individuals with the density *i*(*x*, *t*) recover. Thirdly, individuals infected with a strain *x* may produce a mutant strain *x*′ with a small probability, as explained below (*Mutation*). We measure time in the units of infectious period, *t*_rec_, so that recovery rate is 1, and transmission rate equals the basic reproduction number, *R*_0_, defined as the reproduction number in a population of previously uninfected individuals.

### Mutation

So far we have considered only dynamics of strains *x* which already exist. What drives the antigenic evolution is the emergence of new viral strains. Each strain *x* occasionally and accidentally undergoes a mutation event which changes its ability to be recognized by antibodies (phenotype). We describe this as a variable change in its antigenic coordinate Δ*x* > 0. The new influenza strain with a new antigenic coordinate, *x* + Δ*x*, is either cleared from the individual or (with small probability) transmitted to another person. The model parameters describing random mutations are the average rate *U*_*b*_ per genome per infectious period (Table 1) and the distribution of Δ*x*. The actual distribution may be quite complex [27]; here, we consider a class of exponential distributions [23]. Specifically, we assume that with each mutation, the value of Δ*x* is drawn randomly with the following probability density

$$\begin{array}{c} \rho \left(\Delta x\right)=\frac{e^{-{\left(\Delta x\right)}^{\beta }}}{\Gamma (1+1/\beta )}, \end{array}$$ (2)

where *β* is a fixed parameter.

**Table 1:** Model parameters: Input (upper rows) and output (lower rows).

Notation	Name	Unit	H3N2	H1N1
*R*_0_	Basic reproduction number	1^b^	1.8^a^	1.46^a^
*t*_rec_	Recovery time	day	5^a^	5^a^
*U*_*b*_	Mutation rate per genome	1/*t*_rec_|yr	5 10^−4^|0.036^c^	8 10^−4^|0.058^c^
*a*	Crossimmunity distance	AA	15^c^	14^c^
*K*(*u*)	Crossimmunity function	1	|*u*|/(*a* + |*u*|)	|*u*|/(*a* + |*u*|)
*N*	Population size	1	10^8^	10^8^
*β*	Mutation distrib. parameter	1	2	2
*σ*	Average selection coefficient	1	0.048^d^	0.028^d^
$\frac{365N_{i}}{t_{\text{rec}}N}$	Annual incidence	1/yr	0.07^d^	0.04^d^
*c*	Substitution rate	1/*t*_rec_|yr	0.036|2.6^a^	0.031|2.26^a^
*T*_MRCA2_	Pairwise coalescent time	Year	3.03^a^	4.59^a^

^*a*^ Known from published data for influenza A strains H3N2 and H1N1 [7, 13, 30, 31]

^*b*^ Unit “1” stands for “dimensionless”.

^*c*^ Input parameter of the model which was adjusted to fit published data.

^*d*^ A value predicted for the best-fit parameter set

### Genetic drift

Below in *Results*, we introduce the critically important factor of random genetic drift [28, 29] by allowing the number of new infections to vary randomly among the sources of transmission. The model parameters and their estimates used in the analysis are summarized in Table 1.

## Results

The model described in the previous section establishes a general analytic relationship between immunological, epidemiological, and evolutionary properties of a virus causing non-chronic infection. Using the analytic approach described in the previous section, below we predict the evolution speed, the incidence of influenza in a population, and the time to the most recent common ancestor. Then, we test analytic results with stochastic simulation and compare them to available data on influenza strain A H3N2.

### Recovered individuals and the traveling wave

Below we analyze epidemiological dynamics in two steps. First, we assume that, in the realistic parameter range, *a* ≫ 1, the frequency of infected individuals, *i*(*x*, *t*) represents a solitary peak, much more narrow in genetic distance *x* than the frequency of recovered individuals, *r*(*x*, *t*). Using this fact, we find analytically the form of *r*(*x*, *t*). Second, we apply the well-developed theory of asexual evolution [18–21, 23] to obtain parameters of the distribution of infected individuals *i*(*x*, *t*). Details are given in S1 Appendix; here we present the main steps of the derivation.

We start our analytic derivation by noting that, in the limit of small mutation rates, the main role of mutation is to form new strains with antigenic coordinate *x* larger than for already existing strains. For already existing strains, mutation is negligible. This assumption is intuitively clear and is verified in the relevant parameter range, using estimates of mutation rate *U*_*b*_ (Table 1).

Neglecting the mutation term in Eq 1, we seek for a traveling wave solution of the form *r*(*x*, *t*) = *r*(*x* − *ct*) and *i*(*x*, *t*) = *i*(*x* − *ct*) where *x* − *ct* ≡ *u* is the relative antigenic coordinate of a strain and *c* = d 〈*x*〉/d *t* is the wave speed defined as the average number of non-synonymous nucleotide substitutions per year. Without loss of generality, we choose the peak of the infected wave *i*(*u*) to be at *u* = 0, [d*i*(*u*)/d*u*]_*u*=0_ = 0. The traveling wave solution of Eq 1 for infected and recovered individuals then reads

$$\begin{array}{ccc} i(u) & = & Acf(u),\\ r(u) & \approx & \left\{ \begin{array}{cc} A\exp[-AR_{0}\int_{u}^{0}K\left(v\right)d\;v], & u<0,\\ 0, & u>0, \end{array}\right. \end{array}$$ (3)

where *A* is a constant found from the normalization condition $\int_{-\infty }^{+\infty }[r(u)+i(u)]\text{d}\;u=1$, and *f*(*u*) is a narrow peak with unit area and a width much less than the width of the recovered distribution, *r*(*u*). The wave speed *c* and the shape of the infected density *f*(*u*) are to be determined later on.

At large *R*_0_, *K*(*v*) in Eq 3 can be expanded linearly near zero, so that density of the recovered becomes a half of a Gaussian

$$\begin{array}{c} r\left(u\right)\approx \frac{2R_{0}}{\pi a}e^{-{\left(\frac{R_{0}u}{a\sqrt{\pi }}\right)}^{2}},u<0;\;\;\;0,u>0 \end{array}$$ (4)

and *A* = 2*R*_0_/(*πa*). The fraction of infected individuals in population

$$\begin{array}{c} \frac{N_{\text{inf}}}{N}=\int_{-\infty }^{\infty }i(u)du=Ac=\frac{2R_{0}c}{\pi a} \end{array}$$ (5)

is assumed to be much smaller than 1. Then the annual incidence of infection is expressed in terms of cross-immunity distance, evolution speed, and basic reproduction number as

$$\begin{array}{c} \text{Annual}\;\text{incidence}=\frac{2R_{0}c}{\pi a}\frac{365}{t_{\text{rec}}}, \end{array}$$ (6)

which is a directly testable prediction.

Analytic solution, Eqs 3 and 4, is based on the assumption that the infected wave *i*(*u*) is much more narrow than the recovered wave *r*(*u*). To verify the validity of this approximation, we compare the Eq 3 with Monte-Carlo simulation based on Eq 1. The simulation confirms the existence of a steady traveling wave with two linked components moving to the right in antigenic coordinate (Fig 1). Infected wave *i*(*u*) is, indeed, a narrow peak. The time-averaged solution for recovered individuals obtained from simulation agrees fairly well with the analytic prediction (black line). Recovered wave *r*(*u*) displays a sharp increase near the maximum of *i*(*u*) and a slowly decaying tail at *u* < 0. The sharp increase is due to continuous recovery of infected individuals. The decaying tail is caused by reinfection of recovered individuals once they become genetically remote from the moving front of wave *r*(*u*). This derivation captures only the shape of the recovered peak leaving the narrow infected peak undefined.

### Moving fitness landscape

In order to determine the infected individual distribution, *i*(*u*), we use standard traveling wave theory [18–23]. The interesting feature of the selection due to immune escape is that the fitness landscape which controls the traveling wave travels with the wave. Moreover, it is the wave itself which creates its own landscape, as follows: the recovered create a landscape for the infected evolution, which moves the recovered distribution forward in *x*, and so on.

To derive the form of landscape on the human population level, we use the standard definition of viral fitness as the average number of secondary infections caused by an infected individual [28, 32–34]. (The reproductive number must not to be confused with the basic reproductive number *R*_0_, which is its maximum value, i.e. the value in a totally susceptible population.) Here we choose to define fitness *w*(*x*, *t*) as the log of *R*_0_ − 1, i.e., the exponential expansion rate of the density of infected individuals *i*(*x*, *t*) measured per infectious period:

$$\begin{array}{c} w\left(x,t\right)=\frac{\partial \;\text{ln}\;i(x,t)}{\partial \;t}=R_{0}\int_{-\infty }^{x}K\left(y-x\right)r\left(y,t\right)d\;y-1. \end{array}$$ (7)

**Fig 2:**
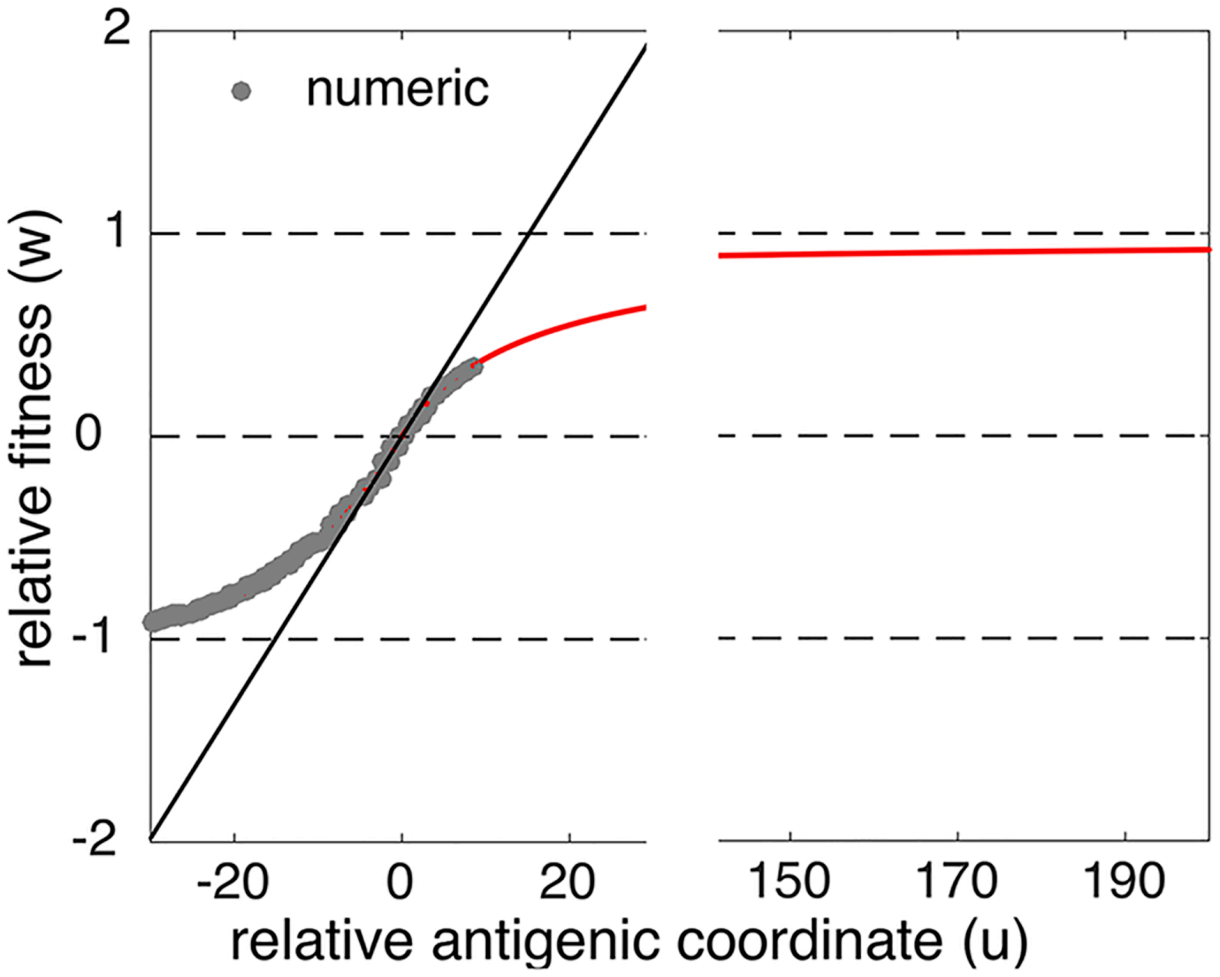
Traveling fitness landscape and its linear approximation near the infected peak. Red curve: analytic result (Eq 7). Gray circles: Monte-Carlo simulation based on Eq 1. Black line: linear approximation with the average selection coefficient *σ* = 0.066 (Eq 8). Parameters as in Fig 1: *R*_0_ = 2, *a* = 9, *U*_*b*_ = 5.8 × 10^−6^, *N* = 10^8^. For the accuracy of linear approximation, see S1 Fig.

The form of *w*(*u*) obtained from Eqs 7 and 3 is shown in Fig 2 (red line).

**Fig 3:**
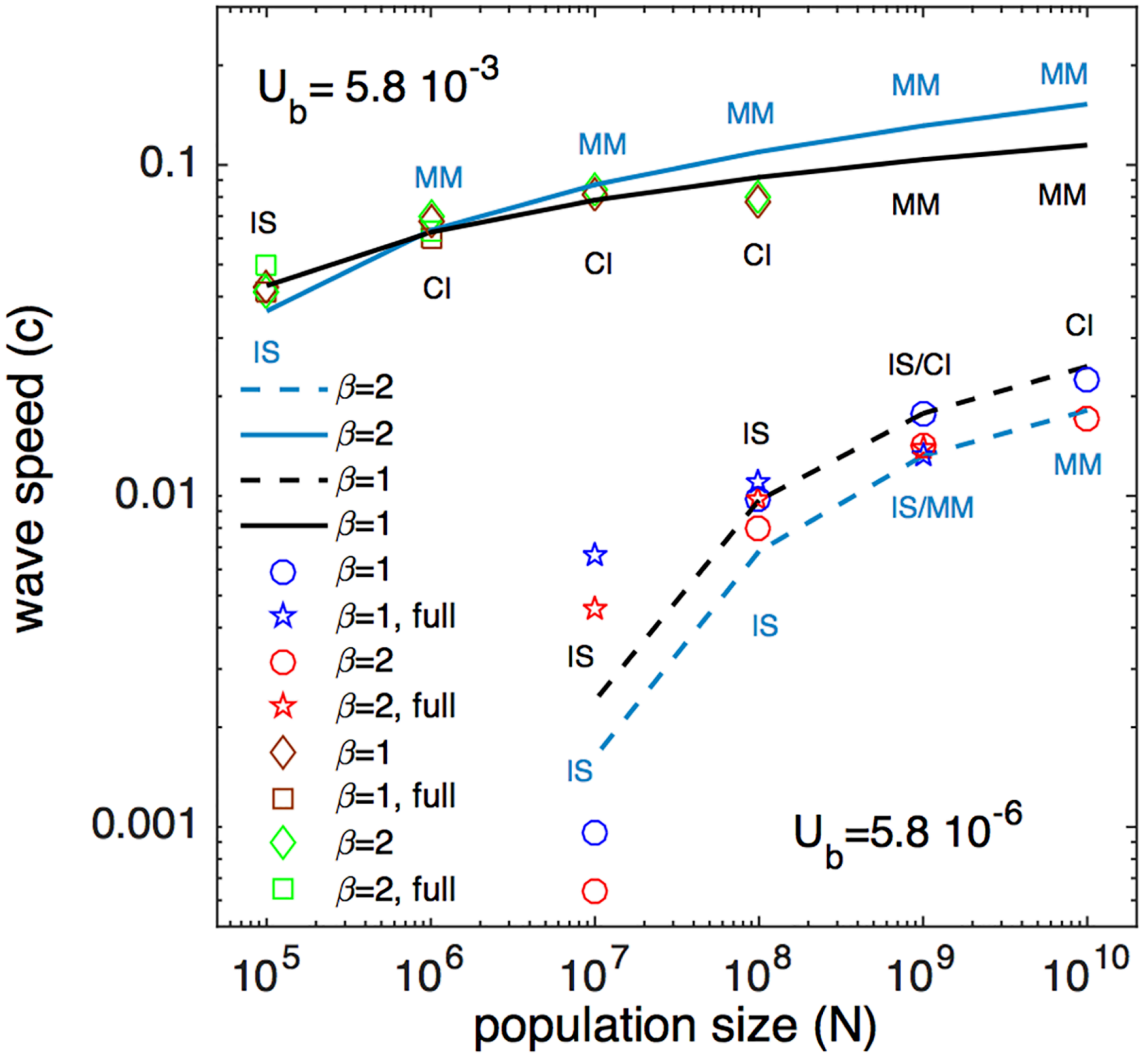
Analytic results for the evolution speed are confirmed by stochastic simulation. Simulation is performed at fixed parameters *R*_0_ = 2, *a* = 9; *U*_*b*_ and *β* as shown. Solid and dashed lines are analytic results for the wave speed, *c* (Eq 6, S14, S16-S18) at two values of mutation rate *U*_*b*_ which define the broadest range of interest for RNA viruses, and two values of parameter *β* to test sensitivity to the density of selection coefficient distribution. Symbols show results either performed by full stochastic simulation of the SIR model (Eq 1) or by a reduced simulation with *σ* = 0.066 (S1 Appendix).

The asymptotic cases of the fitness landscape *w*(*u*) are

$$\begin{array}{c} w\left(u\right)\approx \{\begin{array}{cc} R_{0}-1, & u\gg a,\\ \sigma u,\;\; & |u|\ll a,\\ -1, & u<0,\;\;|u|\gg a \end{array}. \end{array}$$ (8)

where

$$\begin{array}{c} \sigma =-R_{0}\int_{-\infty }^{0}\frac{dK}{du}r\left(u\right)\text{d}u \end{array}$$ (9)

has the meaning of the fitness landscape slope, or the average selection coefficient. According to Eq 8, *w*(*u*) is positive for *u* > 0 and negative for *u* < 0, indicating that viruses are selected for in front of the infected peak and selected against in the wake of the wave. For large positive or negative *u*, |*u*| ≫ *a*, we predict saturation of *w*(*u*). At *u* = 0, *w*(0) = 0, which is equivalent to the fact that the actual reproduction number is exactly 1 at the peak of the wave. Within the range |*u*| ≪ *a*, where the narow peak of the infected individuals is located, fitness landscape can be expanded linearly with slope *σ* > 0 which represents the average selection coefficient of a mutation event. For sufficiently large *R*_0_, from Eqs 4 and 9, *σ* can be approximated by a series in 1/*R*_0_

$$\begin{array}{c} \sigma \left(a,R_{0}\right)=\frac{1}{a}[R_{0}-2+\frac{3\pi }{\sqrt{2}R_{0}}+O(\frac{1}{R_{0}^{2}})], \end{array}$$ (10)

where *a* ≡ 1/|*K*′(0)|, and the second and third terms are small corrections to the first term. Thus, the average selection coefficient *σ* of the traveling fitness landscape is inversely proportional to the cross-immunity distance *a*. It also increases linearly with the basic reproduction ratio *R*_0_ when *R*_0_ is large. The two correction terms in Eq 10 depend on the form of cross-immunity function in Table 1. For an alternative form *K*(*x*) = 1 − exp(−*x*/*a*), they are smaller by factors of 2 and 6, respectively. The overall agreement for the entire landscape *w*(*u*) between the analytic prediction and simulation is quite good (Fig 3).

### Antigenic diversity and the speed of evolution

We get further insight into the dynamics of the model by predicting the speed of viral evolution *c*. So far, we have left this value undetermined because it weakly affects the shape of the density of recovered individuals *r*(*x*, *t*), Eq 3. In contrast, the density of infected individuals *i*(*u*), which is much more narrow, needs to be determined simultaneously with *c*. Our result for the average selection coefficient *σ*, Eq 10, reduces the problem of epidemiological evolution to models of asexual populations with many diverse sites where the speed was derived previously in terms of population size, selection coefficient and mutation rate ([18–23]). We consider a case with randomly distributed selection coefficient *s* = *σ*Δ*x*, where mutational distance Δ*x* is sampled from distribution in Eq 2 with large parameter *β*.

This section contains the central result of our analysis: Antigenic diversity *Var*[*x*] = < (Δ*x*)^2^ > and adaptation rate *v* defined as the average rate of fitness increase (“fitness flux”) depend on crossimmunity range *a* and other parameters [23]

$$\begin{array}{c} Var\left[x\right]=\frac{2\;\text{log}\;N_{\text{inf}}\sigma }{\text{log}(\beta \sigma /U_{b})} \end{array}$$ (11)

$$\begin{array}{c} v=\sigma^{2}Var\left[x\right] \end{array}$$ (12)

Another measure of evolution rate is the average substitution rate *c*

$$\begin{array}{c} c=\left(\sigma^{2}/s*\right)Var\left[x\right] \end{array}$$ (13)

$$\begin{array}{c} s^{*}=\sigma {\left[\frac{\sqrt{2}}{\beta }\;\text{log}\;\frac{\sigma }{U_{b}}\right]}^{\frac{1}{\beta -1}} \end{array}$$ (14)

where s* represents the most probable fitness gain of a mutation established in a population [23]. Note that s* is larger than the average selection coefficient *σ*. The expressions for *Var*[*x*] and s* are approximate, within the accuracy of logarithms inside the large logarithms. For more accurate expressions, see S1 Appendix.

To apply these results to our case of antigenic evolution, we substitute average selection coefficient *σ* from Eq 10 and infected population size *N*_inf_ from Eq 5. Then the metrics of evolution speed *c*, *v* are expressed in terms of *a* and epidemiological parameters (Table 1). In the limit of very large *β*, Eqs 11–14 match results of a model with constant selection coefficient *σ* [18, 20].

We verified analytic results for wave speed *c* by Monte-Carlo simulation in a wide range of *N* and *U*_*b*_ (Fig 3). We used two methods: full simulation of initial Eq 1 with randomly distributed mutational effects, and a reduced Moran algorithm with linearized fitness landscape (symbols in Fig 3). We observe that our analytic prediction of a logarithmic increase of *c* with *N* and *U*_*b*_ follows simulation quite well, except at smallest *U*_*b*_ and *N* explored in our study. Logarithmic dependencies are characteristic for asexual evolution models ([18–23, 35, 36]). Abbreviations IS, CI, MM near symbols indicate different regimes regarding the number of genomic sites evolving within the same time frame: selection sweeps at isolated sites (IS), pairwise clonal interference (CI) [23, 35, 36], and multiple-mutation regime (MM) [18–21, 23]. The traveling wave models are designed for MM regime, which explains the discrepancy at smallest *U*_*b*_ and *N*. We also observe that the steepness of the selection coefficient distribution, *β*, weakly affects the predicted speed.

Our analysis predicts that substitution rate of antigenic mutations *c*, Eq 13, is inversely proportional to the cross-immunity distance *a* and increases logarithmically with host population size and mutation rate. The average selection coefficient at the population level, *σ*, is also inversely proportional to *a*, Eq 10. An alternative measure of the evolution speed, the adaptation rate *v*, Eq 12, is inversely proportional to *a*^2^. The annual incidence of infection, Eq 6 also scales as 1/*a*^2^.

### Time to the most recent common ancestor

Taking advantage of recent theoretical progress in asexual phylogeny [24, 25, 38], we also calculated an important observable quantity, the time to the most recent common ancestor of two co-existing viruses (S1 Appendix, Eqs S20-S21).

$$\begin{array}{c} T_{\text{MRCA2}}=z\sqrt{\frac{2\;\text{log}(N\sigma )}{v}} \end{array}$$ (15)

Here numeric factor *z* depends on the distribution of mutational effect Δ[*x*] [24, 25]. The predicted values are *z* = 1.5 in the case of fixed mutational effect Δ[*x*], and *z* = 3 in the case of the Gaussian distribution of Δ[*x*] (Eq 2 with *β* = 2). Because the Gaussian case is more realistic, and because we are not aware of any results for *T*_MRCA2_ for other forms of distribution, below we choose the value *β* = 2 for data fitting.

### Comparison with influenza A data

**Fig 4:**
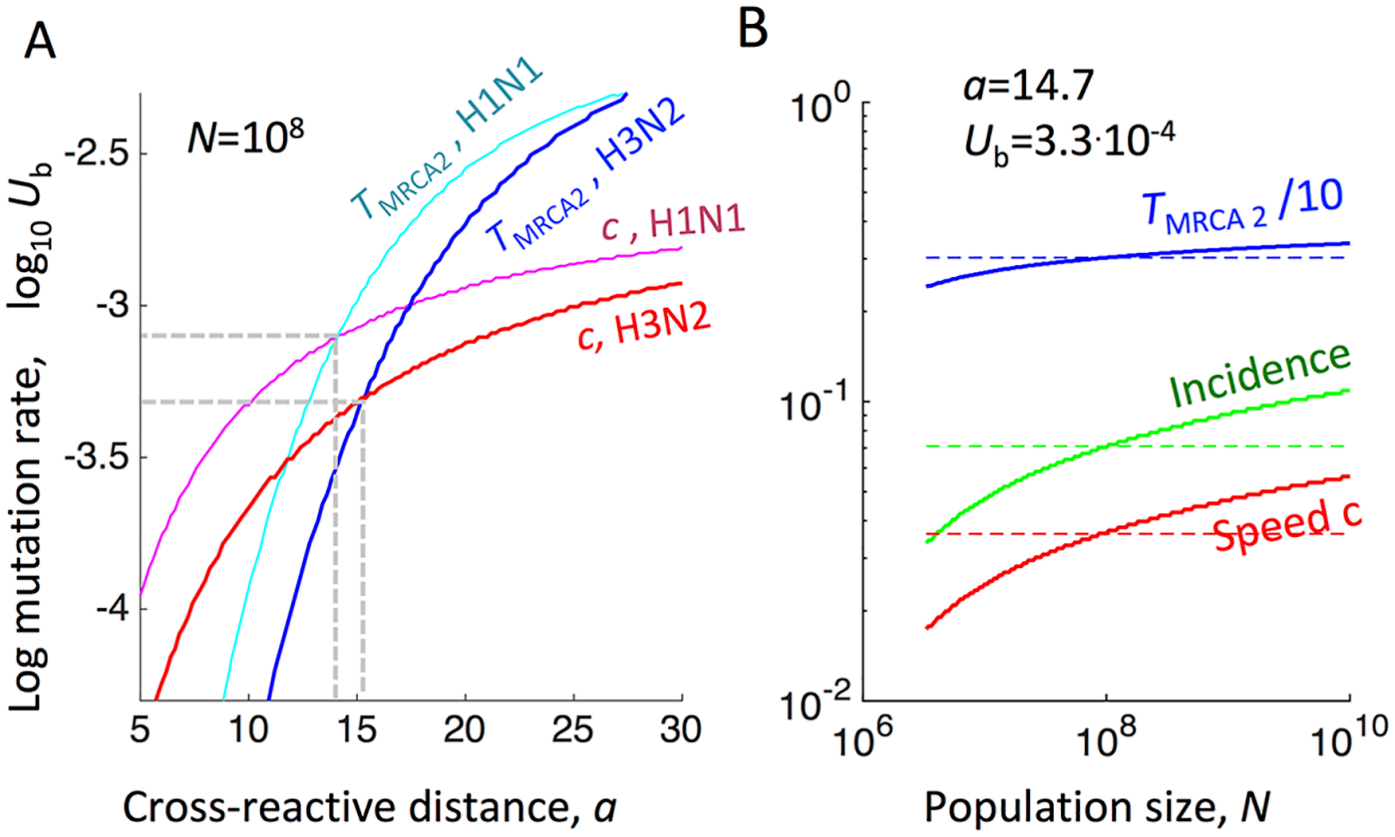
For influenza A virus, the model predicts annual incidence and cross-immunity which agree with observations. Shown is the best-fit to combined immunological, epidemiological, and evolutionary data available on influenza A strains H3N2 (red and blue colors) and H1N1 (magenta and cyan colors). (A) X and Y-axis are the cross-immunity scale, *a*, and the mutation rate per genome per transmission event, *U*_*b*_, respectively. Analytic predictions for the evolution speed *c* (red and magenta curve, Eq 13) and *T*_MRCA2_ (blue and cyan, Eq 15 with *z* = 3) are shown as contours of constant heights taken from data [7] (Extended Data Table 1 and refs). Population size is estimated *N* ∼ 10^8^ [31]. Dashed lines show the intersection points where both parameters fit experimental values. (B) Solid curves: The same three quantities for H3N2 as a function of population number *N* at the best-fit values of *a* and *U*_*b*_. Dashed lines correspond to *N* = 10^8^. (A and B) Input from data [7, 31]: *R*_0_ = 1.8, *c* = 2.6 AA/year, *T*_MRCA2_ = 3.0 years for H3N2 and *R*_0_ = 1.46, *c* = 2.3 AA/year, *T*_MRCA2_ = 4.6 years for H1N1. Infection cycle time *t*_rec_ = 5 days. Predicted annual incidence of infection of (4 − 7)% and the cross-immunity scale *a* = (14 − 15) AA are in good agreement with independent data [37].

To test the model, we compared its predictions with available data on influenza A H3N2 and H1N1, as follows. The input parameters of the model and the output (predicted) parameters are summarized in Table 1. The values of input parameters such as population size *N*, reproduction ratio in the absence of immune recognition *R*_0_ (during a major pandemic caused by antigenic shift), and recovery time *t*_rec_ have been measured [7, 13, 30, 31]. In contrast, parameters *a* and *U*_*b*_ result from biological interactions at multiple biological scales (cell, host, population) and are hard to come by. On the other hand, data on two parameters predicted by the model, *T*_MRCA2_ and substitution rate *c*, are available. Therefore, we opted to adjust the unknown input parameters *a* and *U*_*b*_ to fit available data for the two predicted parameters (Fig 4A). We assumed a total susceptible population of *N* = 10^8^ individuals, which corresponds to a large country.

It is evident that strain H2N3 has a faster evolution rate and a shorter time *T*_MRCA2_ than strain H1N1 due to a larger value of *R*_0_ causing, in turn, a larger average selection coefficient *σ*. The values of *U*_*b*_ and *a* for the two strains are similar (Fig 4a).

The best-fit values for the cross-immunity distance, *a* = 14 − 15, agree very well with independent data on equine influenza [37], which represents a direct confirmation of the model. The predicted annual incidence in humans of (4 − 7)% also falls within the experimentally observed range and previous modeling estimates [12, 13, 15]. Interestingly, the model explains the inverse correlation between *T*_MRCA2_ and evolution rate *c* reported previously for H2N3, H1N1 and two strains of influenza B [7]. Indeed, the predicted evolution rate *c* is linearly proportional to the effective selection coefficient *σ* ∝ *R*_0_/*a*, while *T*_MRCA2_ is inversely proportional to *σ*. The dependence of *c* and *T*_MRCA2_ on the other parameters, *U*_*b*_ and *N*, is logarithmically slow.

To generalize our results for epidemics occurring on larger or smaller scales, we calculated the dependence of *c*, *T*_MRCA2_, and the annual incidence on population size *N* (Fig 4B). The sensitivity of our predictions to input parameters *U*_*b*_, *a*, and *R*_0_ has also been tested (S1 Appendix, S3 and S4 Figs). Thus, traveling wave theory with modest selection predicts logarithmic dependence of the speed on population size (Fig 4B).

### Results are robust to the existence of additional dimensions of antigenic space

Epidemiological data demonstrate that, *a priori*, antigenic space is not one-dimensional but has fractal nature and fractal dimensionality more than 1 [8, 31]. To demonstrate the weak sensitivity of our model to the existence of additional dimensions, we extended our model to a discrete random tree of epitope variants and solved it numerically (S1 Appendix, S6 Fig). Phylogeny demonstrates quasi-1D behavior comprising a long trunk of permanently fixed mutations and short branches representing transient virus variants and resembling the actual influenza H3N2 phylogeny [4, 12, 13, 15]. We also confirmed the formation of a 1D traveling wave for two-dimensional genetic space (S5 Fig).

## Discussion

We investigated stochastic evolutionary dynamics of a virus driven by the pressure to escape immune recognition in previously infected individuals. We mapped this problem to an evolutionary model with fitness landscape expressed in terms of the cross-immunity function *K*(*x*) (Fig 2). Stochastic evolution occurs as a traveling wave with two population components structured in the antigenic variant space *x*, recovered individuals and the currently infected individuals, with different widths and total counts (Fig 1). The recovered distribution is broad and large. The infected distribution represents a narrow and small peak at the recovered distribution front. We expressed several observable parameters including the speed of viral evolution, the annual incidence of infection, and the average time to the most recent ancestor in terms of model parameters *N*, *U*_*b*_, *R*_0_, *K*(*x*) (Table 1). The analytic predictions agree with simulation and are able to estimate correctly important parameters of viral evolution in host populations, as we illustrated using genomic data on influenza.

One of the puzzling aspects of influenza virus evolution is is punctuated nature [4]. While most mutations are almost neutral or have a modest phenotypic (fitness) effect, some represent large jumps in antibody recognition [14]. Our results interpret these jumps as a natural consequence of the stochastic nature of the traveling wave models. The extension of the leading edge of a wave occurs due to adding rare, best available escape alleles. Asexual evolution theory with variable fitness effect of mutations demonstrates that most fixed mutations have a fitness effect in excess of average fitness effect [23]. Good et al show that the most likely selection coefficient s* that drives the wave depends on model parameters *σ*, *N*, *U*_*b*_, mapping the results either onto the multiple-mutation (MM) model with fixed *s* [18–21] or the two-site clonal interference (CI) model [35, 36]. Present work demonstrates that influenza virus evolves within MM regime near the border with CI regime (Fig 3). In this region, the fitness effect of a fixed allele is predicted to fluctuate strongly around the most likely value s*, which represents a possible explanation of the punctuated effect.

An SIR model with immune memory and 1D antigenic space (Eq 1) has been previously proposed by Lin et al [11]. Their analysis differs from ours in two critical aspects. Firstly, their approach to viral evolution was completely deterministic, i.e. assumes infinite population size. In fact, the effect of clonal interference acting in finite population diminishes antigenic return on additional mutations. Secondly, their mutation term in Eq 1 had a diffusion form proportional to the second derivative of the infected individual density, ∂^2^*i*(*x*, *t*)/∂*x*^2^. This approximation would be correct if the front edge of the wave was smooth. As we discuss in S1 Appendix, neither approximation holds at low mutation rates, *U*_*b*_ ∼ 10^−4^. As a result, the approach of Lin et al predicts evolution speeds far below simulation results. The traveling wave approach employed here naturally accounts for both the stochastic effects and the steepness of the leading edge. Future development of this model requires inclusion of finite mutation cost [39].

Our analytic results agree with the numeric results of a previous simulation by Bedford et al [12]. Using a similar model, they predicted the same incidence range for influenza A, the same range for the evolution speed, and interpreted the quasi-one-dimensional trajectory in the genetic space we have also observed (S5 and S6 Figs). As starting parameters, they assumed mutation rate *U*_*b*_ ∼ 10^−4^ and set the cross-immunity distance to be *a* = 1/0.07 based on equine flu data [37]. By comparison, here we determine *U*_*b*_ and *a* a posteriori from fitting human H3N2 and H1N1 data on *c* and *T*_MRCA_ from the cited work [7]. We test the model by comparing our prediction with the experimental value of *a* [37].

## Conclusion

Merging the standard epidemiological approach and the modern traveling wave theory, we develop a general analytic approach that connects epidemiological and immunological parameters to the observed parameters of influenza evolution. We demonstrate that the distribution of recovered individuals in the genetic space effectively creates a fitness landscape for the infected individual distribution, and both distributions move together along quasi-one-dimensional path. Our predictions demonstrate a good experimental agreement with data on influenza A H3N2.

## Supporting information

S1 Appendix Mathematical appendix. (PDF)

S1 Fig Theory of clonal interference with relative fitness linear in antigenic coordinate is accurate at small mutation rates and approximately correct at intermediate rates. (TIFF)

S2 Fig Finite population size N eliminates the artifact of “mirror wave”. (TIFF)

S3 Fig Dependence of the wave speed and incidence on the population size. (TIFF)

S4 Fig Dependence of the wave speed and incidence on the cross-immunity scale. (TIFF)

S5 Fig Two-dimensional influenza model predicts spontaneous development of a stable 1D-like traveling wave starting from a flat front. (TIFF)

S6 Fig Phylogenetic tree of virus strains existing at different times in a multi-dimensional antigenic space projected onto 2D. (TIFF)
