# Correction: Antigenic evolution of viruses in host populations

**DOI:** 10.1371/journal.ppat.1008830

**Published:** 2020-08-12

**Authors:** Igor M. Rouzine, Ganna Rozhnova

In the Time to the most recent common ancestor subsection of the Results, there is an error in the equation. The notation N in parentheses is incorrect. It should read *N*_*inf*_. Please view the complete, correct equation here:

$$T_{MRCA2}=z\sqrt{\frac{2\;\text{log}\left(N_{inf}\right)}{v}}$$ (15)
