# Supplementary material for: Antigenic evolution of viruses in host populations
Source: PLoS Pathog. 2018 Sep 12;14(9):e1007291. doi: 10.1371/journal.ppat.1007291 (PMC6173453; doi:10.1371/journal.ppat.1007291)
Supplement: S1 Appendix — (PDF) [file ppat.1007291.s001.pdf]

Antigenic Evolution of Viruses in Host Populations

Mathematical Appendix

Igor M. Rouzine and Ganna Rozhnova

Contents

Summary
Section 1. 1D model
- 1.1 Model description
- 1.2 Analytic derivation
  - 1.2.1 Solitary wave solution
  - 1.2.2 Traveling fitness landscape and the effective selection coefficient
  - 1.2.3 Wave speed
  - 1.2.4 Time to the most recent ancestor
  - 1.2.5 Comparison to the previous results for a 1D model
- 1.3 Approximations
  - 1.3.1 Linear fitness landscape approximation
  - 1.3.2 Asymmetry of cross-immunity matrix
  - 1.3.3 Single-memory approximation
  - 1.3.4 Regular versus random lattice
- 1.4 Test of analytic theory by numeric simulation

Section 2. Multidimensional models
- 2.1 Two-dimensional models
- 2.2 Tree topology

Section 3. Experimental comparison and parameter fitting

References

Supplemental Figures S1-S6

Summary

Below we consider the evolution of virus variants under the pressure of immune response in a human population. We show how the clonal interference between variants explains the quasi 1D trajectory of influenza, and use predict the speed of evolution, the infection incidence, and the time to most recent common ancestor. We validate the model with data. The work has two parts, with a one-dimensional antigenic space, and a multi-dimensional model justifying the one-dimensional model..

Part 1: 1D model and the Traveling Wave

We describe the dynamics of virus strains by one-dimensional model of genetic variants, corresponding to the main trunk of the phylogenetic tree. The problem is amenable to analytic derivation, which predicts a traveling wave with two components, the susceptible and the infected. The problem differs from the adaptation under constant, externally given selection pressure in that the selection coefficient is determined dynamically, from an SIR model. Below we show how to combine the SIR problem with the dynamic selection to the traveling wave approach (TSIMRING *et al.* 1996; ROUZINE *et al.* 2003; DESAI AND FISHER 2007). These results yield the evolution speed and the incidence of infection, which agree with experimental results. This is the part discussed in the main text..

Part 2. Virus competition for the susceptible individuals forms a quasi-1D trajectory of escape in multi-dimensional genetic space

We consider an N-dimensional genetic space and employ the standard SI approach. The population at any time is comprised of two groups: the infected individuals and the individuals recovered from infections with previous strains and partly susceptible to infection with current strains. Most individuals are those who recovered from strains immunologically similar to the current strain, and only a small fraction individuals have recovered from immunologically distant strains. The currently circulating strains can be transmitted, with full efficiency, only to this last minority of individuals. The competition between virus strains for the most susceptible individuals regulates the reproduction number of the virus in the population in a negative feedback loop, as follows. If, at any time, the reproduction number exceeds 1, and the total infected population expands, the expansion depletes susceptible individuals decreasing the reproduction number below 1. In the long-term, the distribution of strains in the genetic space becomes a system of quasi-1D “snakes” moving on parallel courses (see a two-dimensional example in Fig S5). The snake’s head comprises infected individuals, and the tail comprises susceptible individuals, with the end of the tail harboring the most susceptible. The snakes compete with each other for susceptible individuals until only one survives,

moving on a quasi 1D path. This can also be viewed as a system automatically arriving at a percolation threshold.

1. 1D model

1.1. Deterministic model

We use 1D coordinate *x* to label strains of influenza virus. We define *x* (antigenic coordinate) as the pairwise genetic distance in the antibody-binding region of HA protein between a strain and a reference strain some time in the past. The frequency of individuals in a population infected with strain *x* is denoted $i(x, t)$, and the frequency of the susceptible individuals who previously cleared infection with strain *x* is denoted $s(x, t)$. The susceptible at *x* can be re-infected with a strain $x' > x$, with the efficiency proportional to the cross-immunity function $K(x'-x)$. The half-distance of cross-immunity *a*, defined by the condition $K(a) = 0.5$, is assumed to be large, $a >> 1$. While transmitting, strain *x* can mutate to strain *x*+1 or strain *x*-1 with a small probability U_b ($U_b << 1$). Dynamics of this system is formalized as a system of partial differential equations:

$$\partial i(x,t)/\partial t = i(x,t)\left[R_0\int_{-\infty}^{x} K(x-y)s(y,t)dy - 1\right] + U_b\left[i(x+1,t)+i(x-1,t)-2i(x,t)\right] \quad \text{(S1)}$$

$$\partial s(x,t)/\partial t = -s(x,t)R_0\int_{x}^{\infty} K(y-x)i(y,t)dy + i(x,t) \quad \text{(S2)}$$

The total frequency is one, $\int_{-\infty}^{\infty} dx[s(x,t) + i(x,t)] = 1$.

Below we assume that infection of an individual with strains ahead in the antigenic space of the previously infecting strain is not possible, $K(x<0) = 0$. Also, in this approach, the immune system remembers only the last infecting strain. The role of these approximations is discussed below (Section 1.4). The model in Eqs. S1-S2 is adopted from (Lin *et al.* 2003). Our method and results differ from the cited work substantially (see Section 1.3.4 below).

Note that we measure "antigenic coordinate" *x* in units of genetic distance, defined as the average number of bases that are different in two randomly sampled genomes. Another broadly used unit is the genetic distance causing a two-fold change in HA protein binding by antibodies. We use the first definition, because we is that consider virus evolution on the host population scale, and we define parameters in Table 1, main text, for that scale. In contrast, the HA binding unit are directly relevant on the individual host scale.

1.2 Analytic derivation

In the following subsections, we develop an approximate analytic approach to the problem that applies in the limit of slow evolution, relevant for the real influenza pandemics. First, we demonstrate the existence of a stationary traveling wave solution and predict its shape, without specifying the speed. Then, we derive the time-dependent fitness landscape moving with the wave, and determine the effective selection coefficient. Finally, we apply the traveling wave approach (TSIMRING *et al.* 1996; ROUZINE *et al.* 2003; DESAI AND FISHER 2007) to derive the speed.

1.2.1 Traveling wave solution

Below we assume small wave speeds, $c << 1$, which condition is met in the parameter range of interest (see numeric examples in Fig 4, main text). Then Eqs. S1 and 2 have a traveling wave solution of the form

$$s(x,t) = s[x - x_{\max}(t)], \quad i(x,t) = i[x - x_{\max}(t)], \quad x_{\max}(t) = ct \tag{S3}$$

where c is the wave speed (average antigenic escape rate). Thus, the susceptible and the infected, s and i, each becomes a function of a single variable, specifically, of the relative antigenic coordinate $x - x_{\max}(t)$. Here $x_{\max}(t)$ is the antigenic coordinate of the reference frame moving with the wave. Eqs. S1 and S2 become ODEs

$$-c\frac{di(u)}{du} = i(u)\left[R_0\int_{-\infty}^{u} K(u-v)s(v)\,dv - 1\right] + [\textit{mutation term}] \tag{S4}$$

$$-c\frac{ds(u)}{du} = -R_0 s(u)\int_{u}^{\infty} K(v-u)i(v)\,dv + i(u) \tag{S5}$$

"Mutation term" in Eq. S4 denotes a random variable assuming values 0, $1/N$, $2/N$, with the average given by the last term in Eq. S1. The mutation term is small (due to $U_b << 1$), and we can safely neglect it for most values of u. Mutation becomes critically important at the long ($u >> 1$) leading edge of the wave, where new most-fit virus variants are generated (see subsection *Wave speed* below).

Without the loss of generality, we can define coordinate u so that the peak of infected individual density $i(u)$ is at u=0, as given by $[di/du]_{u=0} = 0$. Setting u=0 in Eq. S4 and neglecting the small mutation term, we have

$$R_0\int_{-\infty}^{0} K(-u)s(u)\,du = 1 \tag{S6}$$

which reads that the effective reproduction number at the peak at u=0, by definition, is equal 1.

Now, we derive the frequency of the susceptible individuals $s(u)$ from Eq. S5. As we verify at the end of this subsection, $i(u)$ has the width $u \sim 1$. Eq. S6 implies that the

width of $s(u)$ is large, $|u| \sim 1/a >> 1$. Therefore, $i(u)$ in Eq. S5 can be approximated with a delta-function

$$i(u) \approx Ac\delta(u) \tag{S7}$$

where A is a constant. Product $cA = \int_{-\infty}^{\infty} dx\, i(x,t) \ll 1$ is the prevalence of influenza in a population, which is known to be small (Table 1). Taking into account Eq. S7, Eq. S5 takes the form

$$ds/du = AR_0 s(u)K(-u) - A\delta(u), \quad u < 0 \tag{S8}$$

Integrating Eq. S8 with the boundary condition $s(-\infty) = 0$ and using Eq. S6, we obtain

$$s(u) = \begin{cases} A\exp\left[-AR_0\int_0^{-u} dvK(v)\right], & u < 0 \\ 0, \quad u > 0 \end{cases} \tag{S9}$$

Here constant A is determined from the normalization condition

$$\int_{-\infty}^{\infty} s(u)du = 1 - \int_{-\infty}^{\infty} i(u)du = 1 - Ac \approx 1 \tag{S10}$$

Eq. S9 implies that the characteristic width of $s(u)$ is on the order of a, as we already obtained before. Thus, the narrow peak of the infected at u=0 is followed by a gradually decaying tail of the susceptible (Fig 2A, main text).

1.2.2 Traveling fitness landscape and the effective selection coefficient

To link our problem to the traveling wave theory and calculated the wave speed c, we need to interpret Eq. S1 in terms of fitness. The standard definition of virus fitness is the reproduction number, i.e., the average number of individuals infected by an individual (RICE 2004; NOWAK 2006; ASTIER 2007; POULIN 2007). It is more convenient to define fitness $w(u)$ as the logarithm of this quantity equal to the exponential expansion rate (the time unit is one generation), which represents the average fitness effect of a mutation event

$$\Delta[w(u)] \equiv \frac{\partial \ln i(x,t)}{dt} = -\frac{1}{c}\frac{\partial \ln i(u)}{du} = R_0\int_{-u}^{\infty} K(v+u)s(-v)dv - 1 \tag{S11}$$

where $s(u)$ is given by Eq. S9. Fitness landscape $w(u)$ and its linear expansion are shown in Fig. 3. We can simplify Eq. S11 in three asymptotic limits, as follows:

$$w(u) \approx \begin{cases} R_0 - 1, & u >> a \\ \sigma u, \quad \sigma \equiv R_0 \int_0^\infty K'(u) s(-u) du, & |u| << a \\ -1, & u < 0, |u| >> a \end{cases} \tag{S12}$$

To obtain the first case, we approximated $K(v+u)$ in the integrand of Eq. S11 with its asymptotic plateau value at large positive $v+u$, 1. In the second case, we replaced the RHS of Eq. S11 with its linear expansion in u. In the third case, we neglected the integral in Eq. S11 because the overlap between $K(v+u)$ and $s(-v)$ is small.

Thus, the fitness landscape travels with the wave. Virus strains are selected for in front of the infected peak and decline in the wake of the wave. Parameter σ represents the fitness gain per mutation for strains located near the infected peak ($u = 0$) and plays the role of selection coefficient in the standard traveling wave theory. The value of σ in Eq. S12 is small: from the scaling of $K(u)$ and $s(u)$ implied in Eqs. S9 and S10, we have $\sigma \sim 1/a << 1$. Replacing fitness landscape with its linear expansion leads to accurate results whenever the leading edge of the wave stays confined within boundaries of the linear regime (see Section 1.3.1 and Fig. S1). To increase the accuracy of the analytic predictions in the entire range of parameter values explored in our study, we also calculated the corrected value of σ that accounts for the nonlinear behavior (Eq. S23).

1.2.3 Wave speed

The evolution speed is defined biologically as the wave speed in terms of nucleotide substitutions, $c = dE[x_{\max}(t)]/dt$ and mathematically in Eq. S3. So far, we left the value of c undetermined, because we considered only the distribution of the susceptible individuals s in antigenic coordinate. We showed that the form of that distribution is not affected by c when c is small.

In the present subsection, we calculate the wave speed (evolution rate). For this purpose, we will include the standard traveling wave theory (Tsimring *et al.* 1996; Rouzine *et al.* 2003; Desai and Fisher 2007) into our analysis. This will also allow us to compare the predicted rate of antigenic evolution and the average annual infection incidence with experimental data below. As the cited models show, generally speaking, the speed of asexual evolution cannot be found from a purely deterministic consideration. Linkage and finite population size N result in competitive interference between advantageous mutations randomly arising at different sites, which effect decreases the speed.

So far, we used the fact that, at small mutation rates, the width of the distribution of the infected individuals, $i(u)$, is narrow compared to the width of susceptible ($|u| \sim a$). Therefore, we approximated $i(u)$ with a delta-peak. Now we have to address the width and the shape of $i(u)$. According to the standard theory, the shape of $i(u)$ is

approximately Gaussian in the center, and has exponentially decaying tails, of which especially important is the leading tail (u > 0). Due to finite (but still very large) population size N, the real distribution of infected individuals $i(u)$ ends at the leading edge at some value u_0 >> 1. Rare stochastic events of generation and establishment of new best-fit genomes at the leading edge $u = u_0$ limit the speed of the wave progress.

Below we relate the speed c to the population size N, the genomic mutation rate U_b, and the average selection coefficient σ (Eq. S11) using the analytic "traveling wave" approach (TSIMRING *et al.* 1996; ROUZINE *et al.* 2003; DESAI AND FISHER 2007). The expression for c varies depending on the distribution of selection coefficient $\sigma\Delta[x]$ among individual mutation events, as follows.

The case of fixed selection coefficient. In the simplest case of fixed σ, the speed c is a function of population size, U_b and s, as found from the transcendental equation [(ROUZINE *et al.* 2008), Eq. S52]

$$\ln N_{\text{infec}} = \frac{c}{2\sigma}\left(\ln^2\frac{c}{eU_b}+1\right) - \ln\sqrt{\frac{\sigma^2 U_b}{c\ln\left(c/U_b\right)}} \qquad \text{(S13)}$$

where

$$N_{\text{infec}} = cNA \qquad \text{(S14)}$$

is the total number of infected individuals, N is the total number of individuals, and A is given by Eq. S9. The edge location with respect to the wave peak, u_e, is given by [(ROUZINE *et al.* 2008), Eq. S39]

$$u_e = \frac{c}{\sigma}\ln\frac{c}{eU_b} \qquad \text{(S15)}$$

Exponential distribution of selection coefficient. In real systems, σ varies randomly among sites. It is more convenient to consider other measure of the evolution speed than substitution rate c, namely, the adaptation rate r defined as the linear rate of fitness gain with respect to the virus that can mutate (U_b = 0). When σ is distributed exponentially with the average σ_{av}, as given by Eq. 2 in the main text with β=1, the adaptation rate v *defined as the average rate of fitness increase per unit time* is found from the coupled equations for v and a new composite parameter w_e [(GOOD *et al.* 2012), Eqs. S14-S15]

$$2 = \frac{U_b}{\sigma}\sqrt{\frac{2\pi\sigma^2}{v}}\left[1+\frac{v}{w_e}+\frac{v}{\sigma w_e - v}e^{\frac{\left(w_e-\frac{v}{\sigma}\right)^2}{2v}}\right] \qquad \text{(S16)}$$

$$1 = N_{\text{infec}}U_b\left[-1+\frac{(w_e)^2}{v}+\frac{2\sigma\,(w_e+\sigma\,)}{v}\right]e^{-\frac{2\sigma w_e - v}{2\sigma^2}} \qquad \text{(S17)}$$

Here the new parameter w_e is defined as the fitness lead of the wave, the distance between the high-fitness edge of the traveling wave and its peak. By definition, of w_e, fixation of newly emerging beneficial alleles with larger fitness effect, $w > w_e$, is not affected by the clonal interference with the rest of genome. Such alleles are fixed with probability w, as in a independent-site model (the limit of strong recombination). The substitution rate c is found as

$$c = \frac{v}{s^*}, \quad s^* \equiv w_e - \frac{v}{\sigma} \qquad \text{(S18)}$$

The biological meaning of s^* is the most probable value of fitness effect σ of mutations that are fixed in a population.

To predict the substitution rate, c, the system of Eqs. S16 and S17 has to be solved numerically (main text, *Results*). In the main text, the predicted values of c are compared to simulation results in a range of parameters a and N (Fig 3, main text). The annual incidence of infection $(365/\tau_{\text{inf}})\, Ac$ (Eq. S9) agrees with the observed value (Fig. 4, main text).

Arbitrary distribution of selection coefficient. Good et al (GOOD *et al.* 2012) considered a more general case, in which the distribution density of fitness gain σ has an arbitrary form $\rho(\sigma)$ (all mutations are beneficial, $\sigma > 0$). The adaptation rate v and the fitness edge w_e are found from the coupled equations al [(GOOD *et al.* 2012), Eqs. 17-19]

$$\begin{aligned}
& s^* - w_e - v\frac{\partial \log \rho(s^*)}{\partial \sigma} = 0 \\
& 2 = \frac{U_b\sqrt{2\pi\Delta^2}\rho(s^*)}{s^*}\left[1 - \frac{s^*}{w_e}\right]^{-1} \exp\left[\frac{w_e s^*}{v} - \frac{s^{*2}}{2v}\right] \\
& 1 = \frac{2Ns^*}{\sqrt{2\pi}}\sqrt{\frac{w_e^2}{v}}\exp\left[-\frac{w_e^2}{2v}\right] \\
& \Delta^2 \equiv \left[\frac{1}{v} - \frac{\partial^2 \log \rho(\sigma)}{\partial \sigma^2}\right]^{-1}_{\sigma = s^*}
\end{aligned} \qquad \text{(S19)}$$

The speed c and annual incidence $(365/\tau_{\text{inf}})\, Ac$ are given by Eqs. S18 and S9, respectively. For the case $\rho(\sigma) \propto \exp(-(\sigma/\sigma_{\text{av}})^2)$, $\sigma > 0$, which corresponds to β=2 in Eq. 2 of the main text, we plot the value of c in Fig. 3, main text.

The case of large β. In the case $\beta >> 1$ (distribution in Eq. 2 in the main text), Eqs. (S19) simplify as

$$w_e{}^2 \approx 2v\log(N\sigma)$$

$$v = \sigma^2 Var[x] = \sigma^2 \frac{2\log(N\sigma)}{\log\left[\frac{\sigma}{U_b}\right]}$$

$$s^* = \sigma\left[\frac{\sqrt{2}}{\beta}\log\frac{\sigma}{U_b}\right]^{\frac{1}{\beta-1}}$$

$$c = v/s^* \qquad \text{(S20)}$$

<u>Simulation.</u> We test our analytic results using two different methods of stochastic simulation. One is a full Monte-Carlo simulation of the SIR model based on Eqs. 1 in the main text including the stochastic mutation term. This simulation predicts time dependencies for a set of antigenic coordinates $x_i(t)$ corresponding to virus strains in infected individuals. The second method is a continuous-time Moran simulation of infinite-site model with a selection coefficient σ_k assigned to each string position k and drawn from a random distribution of an exponential form (Eq. 2 in the main text). This method is similar to that used by (GOOD *et al.* 2012), Either simulation generate a time-dependence of the average infected individual density $i(x,t)$. As a result, we predict that the evolution speed c and the infection incidence are weakly sensitive to the shape index of the exponential distribution, β (Fig. 3, main text).

1.2.4 Time to the most recent ancestor

An accurate prediction for the average time to the most recent common ancestor of a pair of individual genomes T_{MRCA2} for an arbitrary distribution of σ has not been published. However, analytic predictions for the fixed-σ model and a model with a Gaussian distribution of σ symmetric around 0 have been derived, and simulation tests have been done (WALCZAK *et al.* 2012; NEHER AND HALLATSCHEK 2013). Results of these two works can be approximated as follows:

$$T_{\mathrm{MRCA2}} = \frac{z w_e}{v} = z\sqrt{\frac{2\log(N\sigma)}{v}} \qquad \text{(S21)}$$

where w_e/v given by Eq. S20 represents the time in which the wave moves by the distance between its maximum and high-fitness edge, and z is a numeric factor on the order of 1. Specific choice of z depends on the form of selection coefficient distribution $\rho(\sigma)$, Eq. 2 main text, as follows

$$z = \left[\begin{array}{ll} 1 & \sigma = const, \sigma > 0, \text{ analytic} \\ 1.5 & \sigma = const, \sigma > 0, \text{ simulation} \\ 3 & \beta = 2, \text{ simulation} \end{array}\right. \qquad \text{(S22)}$$

Note that simulation shows a larger value of T_{MRCA2} than the analytic derivation in the relevant parameter range. The evolution of HIV and influenza involves mutations with both positive and negative sign of σ (BATORSKY *et al.* 2014; LUKSZA AND LASSIG 2014).

In the main text, we compare the predicted value of T_{MRCA2} with available data for influenza A H3N2 (Fig 4, main text). Comparison with data on T_{MRCA2}, Because we do not know the exact form of the distribution of $\rho(\sigma)$, in Eqs. S21 and S22, we approximated our situation with the case β=2, in which case z=3. This case is more realistic than constant mutation fitness effect.

To calculate the speed c in the main text for Fig. 4 more accurately, we used Eqs. S20, but replaced log(Ns) with

$$\ln\left[N\sqrt{\frac{\sigma^2 U_b}{c\ln\left(c/U_b\right)}}\right]$$

i.e., we used the more accurate pre-factor at N from Eq. S13 obtained originally for the case of constant s.

1.2.5 Comparison to a previous 1D model of influenza evolution

A similar 1D model of the influenza pandemics was proposed in the pioneering work by Lin et al (LIN *et al.* 2003). These authors predicted a traveling wave solution and calculated its speed. Their method differs from ours, both conceptually and quantitatively, in two crucial aspects:

(i) Their model is based on Eqs. S1 and S2, where the mutation term has the diffusion form proportional to a second derivative in x.
(ii) Infinite population size is assumed.

Neither approximation holds in the experimentally relevant parameter range (Table 1). The diffusion approximation of mutation applies, and the difference between x and x+1 in Eq. S1 can be replaced with the 2nd derivative, only if the decay of the wave at leading slope is gradual, as given by $|d\ln i(x,t)/dx| << 1$. According to Lin et al's asymptotic solution [(LIN *et al.* 2003), Eq. S16], this would apply if $2U_b >> R_0-1$. (The condition is given in our notation. In the cited work notation, it is $\lambda/a = c/(2ad) << 1$.) This is clearly not true in the actual data sets where the mutation rate of influenza virus U_b is very small, and the reproduction ratio R_0 is on the order of one (Table 1). Thus, the leading front of the infected wave is in fact steep and the cited solution is not self-consistent. Also, finite population size is important up to extremely large population sizes (ROUZINE *et al.* 2003). Both factors are naturally taken into account in the traveling wave approach.

Consequently, the cited results differ from the results of stochastic simulation obtained in the relevant parameter range (Table 1). For the evolution speed (LIN *et al.* 2003) predict [see equations in their work: Eq. S3, S12, and the equation before S3]

$$c_{\mathrm{Lin}} \approx \frac{2}{a}\sqrt{2U_b(R_0-1)}\left[1-\frac{\pi^2}{2\ln(N)^2}\right] \qquad \text{(S23)}$$

which is an order of magnitude below the simulation results in Fig 3, main text. Still another difference is in the scaling of the widths of infected and susceptible distributions in genetic distance with the mutation rate U_b. (LIN *et al.* 2003) predict the scaling $U_b^{1/2}$ and $U_b^{1/4}$, respectively [(LIN *et al.* 2003), Fig. 3]. In both our simulation and analysis, the susceptible distribution width does not depend on U_b, and the infected distribution width depends on U_b logarithmically. In agreement with Fisher's Fundamental Theorem and Eq. S13, it is proportional to the substitution rate, c.

1.3 Approximations

The analysis is based on three approximations as follows: (i) Replacing the fitness landscape with its linear expansion (the selection coefficient approximation), (ii) prohibiting infection with virus strains ahead of the wave (the asymmetry of the cross-immunity matrix), and (iii) accounting for the immune memory of the last infecting strain only (the single-memory approximation). Below we show that, in the relevant parameter range, approximation (i) somewhat limits the accuracy, but is still practically useful for the experimental comparison. Approximation (iii) may cause instability in the far trailing tail of the wave, an artifact compensated by (ii).

1.3.1 Linear fitness landscape approximation

To calculate the rate of evolution, we adopted the results of traveling wave by approximating fitness landscape with its linear Taylor expansion (second line in Eq. S12). The actual fitness $w(u)$ is predicted to saturate at large negative and positive u (Fig 2). The saturation at large u may become critical at the leading edge of $i(u)$. The traveling wave theory [(ROUZINE *et al.* 2003), (ROUZINE *et al.* 2008), Eq. S39] predicts the edge location ($u = u_0$) with respect to the peak location ($u = 0$) (Eq. S14). For the linear approximation to be approximately valid, the leading edge should stay within the linear range.

Numeric simulation predicts a similar edge location as the analytic theory. Showing the edge location against the fitness landscape (Fig S2, vertical dashed lines), we can observe that the linear approximation is accurate at reasonable mutation rates $U_b = 10^{-6}$ -10^{-4} and even at very high rates $U_b = 5{\cdot}10^{-3}$ it overestimates the fitness slope at the leading edge only by factor of 2 (at realistic N). The deviation from linear fitness landscape create an overshoot of analytic predictions as compared ti simulation results for speed c at large N and U_b (results not shown).

To partly compensate for the nonlinearity, we repeated the derivation given in (ROUZINE *et al.* 2008) (see their mathematical *Supplement*) for arbitrary fitness landscape $w(u)$. The final result has an approximate form of Eq. S13, but with u_0 and σ determined from

$$w(u_0) = c \ln \frac{c}{eU_b}$$
$$\sigma = \frac{w(u_0)^2}{2\langle w \rangle_{u_0} u_0} \tag{S24}$$

which replace Eq. S15 and the 2nd of Eq. S12, respectively. Here $\langle w \rangle_{u_0}$ is the average fitness over the interval $0 < u < u_0$. The new effective value of σ accounts for the non-linear behavior. Analytic results shown in main text (Fig 3) are corrected for this effect.

1.3.2 Asymmetry of cross-immunity matrix

The analysis so far assumed an asymmetric immunity matrix, $K(u<0)=0$ (Eqs. S2 and S3). Briefly, strains u were not allowed to infect the individuals recovered from infections with strains lying ahead in the antigenic coordinate, $u' > u$. Mostly, the approximation does not affect our results, because there are no susceptible individuals far ahead of the infected peak (Fig. 1, main text). However, the restriction may actually help in the far trailing tail of the wave, where it compensates for an instability artifact. If the immunity function is symmetric then there is a parameter range where a second wave may emerge from the trailing tail of the main wave and start rolling backwards (Fig S3A). Either cutting off the distribution at finite $1/N$, Fig S3B, or prohibiting infection forward in fitness eliminates this problem without affecting any other results.

To explain this phenomenon analytically, let us consider for a moment a symmetric immunity matrix $K(u)$, such that $K(u) = K(-u)$. In this case, the upper limits in integrals in Eqs. S4 and S6 are replaced with infinity, and the lower limits in Eqs. S5, 9, 11, and 12 with negative infinity. Because $i(u)$ is still a very narrow peak in u from the perspective of the immunity scale $|u| \sim a$, the shape of $s(u)$ is not affected, and most of the results apply, except for a change in the trailing end of the wave u, $|u| >> a$. The new fitness landscape replacing Eq. S11 now has a form

$$w(u) = R_0 \int_{-\infty}^{\infty} K(v+u)s(-v)dv - 1 \tag{S24}$$

As it follows from Eq. S24, fitness $w(u)$ is no longer a monotonous function of u that was shown in Fig. 2, main text, and Fig. S1. As u decreases, instead of saturating at -1, as in Fig 3, main text, $w(u)$ goes through a flat minimum and then increases to become again at $|u| >> a$, $w(-\infty) = R_0 - 1$ (the plot not shown). In other words, as the wave moves over, extremely low counts of old strains left in a population start to

grow again. As a result, a new peak appears in the trailing edge and moves backward, towards negative u (Fig S3A). This mirror wave is not a biological effect, but a mathematical artifact of the single-memory approximation employed in the text. At modest population sizes $N \sim 10^6$ - 10^8 or large $a > 15$, the artifact is eliminated by the cutoff of the tail at a single individual [at $s(u) \sim 1/N$] already included in our simulation. An alternative method which works in the entire parameter range, is to prohibit the infection forward by choosing an asymmetric immunity function $K(u)$, as we did everywhere in the text. At moderately large a, this asymmetry of $K(x)$ has no other effects on the evolutionary dynamics in 1D.

1.3.3 Single-memory approximation

The backward wave is an artifact of the single-memory approximation introduced to make the problem analytically tractable. We assumed that an individual has only memory cells from the last infection. This is a simplification. In reality, the immune system of an individual "remembers" not only the last infecting strain u, but all the previous infections (JANEWAY AND TRAVERS 1996). The single-memory approximation we employ creates cycles of reinfection within a small group of strains, such as $x_1 \to x_2 \to x_1$. The backward wave in 1D is an example of such a reinfection loop.

In the absence of the artifact of the backward wave, the single-memory approximation is valid at large a, because the consecutive strains that a typical individual is infected with are sufficiently far apart. Indeed, the average last infecting strain is at the distance $u = -a$ behind (Fig. 1 in the main text). Therefore, the earlier strains (the memory of which we neglected) are located at average coordinates forming the arithmetic progression $u = -2a, -3a, -4a, \ldots$. At these remote locations in the left tail of the susceptible individuals (Fig 1, main text), they are very few compared to the maximum at the edge u=0. Therefore, the approximation has a weak effect on results.

1.4 Numeric tests

We test our analytic results using two different methods of stochastic simulation. One is a full Monte-Carlo simulation of the entire SIR model including Eqs. S1, S2 and stochastic mutation, with individual binary strings and random fitness effect of mutation. This simulation predicts time dependencies for a set of antigenic coordinates $x_i(t)$ corresponding to virus strains in infected individuals. The second method, designed to test the evolutionary component of the model, is a more restricted continuous-time (Moran's algorithm) simulation. First, we calculated the effective selection coefficient s_k from Eq. S1 and S2. Then, we simulated the infinite-site model which assumes that mutation never happens at the same site more than once. Fitness effect of each mutation occurring at random time was drawn from a random distribution. Either type of simulation generated very similar time-dependence of the average infected individual density $i(x,t)$, fairly similar to the analytic results (Fig. 1, main text).

<u>Section 2. Multidimensional models</u>

Then we tested whether a 1D model of genetic space is adequate for evolution of immune escape. For this end, we considered two generalizations of the 1D model: a two-dimensional lattice model (Fig. S5) and a random tree model with p neighbors per node with random component (Fig. S6). Results demonstrate that a 1D wave emerges automatically in either case due to random landscape and competition for resources. Thus we demonstrate that main conclusions are robust to the presence of multiple dimensions.

2.1 Two-dimensional genetic space (Fig S5)

One antigenic, one neutral coordinate. In the two-dimensional model, we consider a 2D square lattice $\{i, j\}$. We define the antigenic coordinate $x_{ij} = i + \xi_{ij}$ and the neutral coordinate $y_{ij} = j + \eta_{ij}$, where ξ_{ij} and η_{ij} are random values uniformly distributed between $-V_x$ and V_x. The antigenic coordinate can be chosen arbitrary along any line on the plane but for simplicity we consider the x-axis. (We also turned it by 45 degrees without substantial change in results.) The antigenic coordinate for a strain is a sum of two terms — a regular term equal to the number of the lattice point (in the horizontal direction) and a random contribution drawn uniformly from $(-V_x, V_x)$, where V_x is a parameter additional to the one-dimensional model. The second coordinate of a strain in this model is neutral and does not influence its antigenic properties.

Immunity matrix is defined between each two lattice points as $K_{ij,mk} = K(x_{ij} - x_{mk})$, where $K(u < 0) = 0$ is the same as in the 1D model (Table 1, main text). (We also considered a symmetric case, see below.) Dynamic equations for each lattice point $\{i, j\}$ are given by discrete versions of Eqs. S3 and S4, in which the integrals are replaced by the double sums over the lattice vertices. Mutation can occur in all four directions, with the same rate per site U_b.

Several snapshots of the dynamics of the two-dimensional model (Fig S5) demonstrate the spontaneous development of a steady 1D-like wave from a flat front. Starting from the delta distribution for the infective density (brown strip in the top left panel) and a uniform distribution for the susceptible density (brown rectangle in the bottom left panel) the system develops several competing 1D-like waves. Due to the randomization of the antigenic coordinate the head of one of the infective waves happens to be further in the antigenic coordinate which facilitates the infection of the susceptible in the tail of the other waves (middle rows). This competition between different infective waves for the common pool of the susceptible ultimately leads to a single wave shown in the right-most panels of the figure. The investigation of the model's dynamics leads to the conclusion that the typical width of a wave in the horizontal direction is determined by the parameters of the one-dimensional model, particularly a and R_0, and that in the vertical (neutral) direction is proportional to V_x.

Numeric simulation is, as follows. We start from a straight strip of the infected along the left side of the square, with the susceptible filling the strip behind (Fig S5, left panels). As the infected wave moves forward, it curves randomly. Tongues shoot forward, their number decreases, and finally, a single island is left, with a tail of the susceptible trailing behind. The system resembles a system of 1D snakes (comets) moving on parallel courses (Fig S5, center panels). The head of each snake is the infected individuals, and the tail is the susceptible. The mechanism behind this dynamics is that different strains compete for a limited resource, the susceptible individuals. As a result of this competition, there exists a back feed from the infected to the susceptible distribution, which sets the branching ratio to 1 and brings the random system to the percolation threshold.

Two antigenic coordinates. To test whether the assumption of a single antigenic coordinate is restrictive, we also performed simulation in an anisotropic 2D model with two asymmetric antigenic coordinates (x, y). The cross-immunity matrix depending on the elliptical Euclidian distance in the plane

$$K_{ij,mk} = K\left(\sqrt{\left(x_{ij} - x_{mk}\right)^2 + asym * \left(y_{ij} - y_{mk}\right)^2}\right)$$

where $asym < 1$ is the asymmetry factor. Results confirmed the appearance of a quasi-1D wave, although with some complications, such as appearance of a wake and a back wave, likely to be artifacts of the single-memory approximation (Section 1.3.3).

2.2 Tree topology of genetic space (Fig S6).

We also performed Monte-Carlo simulations using tree topology for parameter sets where we varied U_b, N, R_0, a and V_x. In this case, the antigenic coordinate of a strain was defined as $x = x_f$ + a random contribution drawn uniformly from $(-V_x, V_x)$, where V_x is a parameter additional to the one-dimensional model and x_f is the antigenic coordinate of the parental strain. The total antigenic distance between two nodes i and j was added along the (single) path connecting these two nodes. The cross-immunity function K_{ij} was asymmetric and depended on the total distance as in the 1D model (Table 1, main text). Model parameters were $U_b = 0.001$, $N = 10^8$, $R_0 = 3$, $a = 10$. The antigenic coordinate of a strain was set to be $x = x_f$ + [*random number between* 0.5 *and* 1.5].

An example of time-dependent phylogenetic tree is shown in Fig. S6. Thus, a quasi-1D trajectory arises automatically from either 2D or a tree topology (Fig. S5 and S6) . These results demonstrate that the main results of the present work are not restricted by the 1D model used for the main part of analysis.

Section 3. Experimental comparison and parameter fitting (Table 1 and Fig. 4, main text)

To estimate parameters and verify the 1D model, we fit our model to data, as follows. In the epidemiological literature and on CDC site, the annual incidence of influenza A (H3N2) varies in the range of 1-15% of annually infected individuals. The average evolution rate and T_{MRCA2} are known more accurately, c=0.036 aminoacid substitutions/genome/cycle (1 cycle = 5 days) and T_{MRCA2} = 3.03 years [see refs in Figure 4 caption]. The population size relevant for influenza (which is required only within logarithmic accuracy) is on the order of $N=10^8$-10^9 individuals. Immunity-free reproduction ratio R_0=1.8 can be approximated by its value determined from the most rapid pandemics, such as those that occurred in 1918 and 1968.

To compare our analytic model with data, we used mutation rate U_b and cross-immunity distance a as fitting parameters. The effective epitope length defined as the total number of mutating aminoacids within the antibody-binding regions of HA protein spans L=120 non-synonymous nucleotides (~60 aminoacids) (LASSIG 2012; LUKSZA AND LASSIG 2014). The mutation rate per nucleotide per infectious period was estimated from the synonymous substitution rate, $5.8 \cdot 10^{-5}$ (LASSIG 2012; LUKSZA AND LASSIG 2014). However, not all aminoacids participate equally in the host B cell immune response, and not all mutations of the immunologically relevant aminoacids abrogate immune recognition by a value close to 100%. Therefore, the effective epitope length is expected to be below 60. It is difficult to determine from any direct experiment or observation. Therefore, the immunologically relevant non-synonymous mutation rate per genome U_b is usually estimated roughly (guessed) (BEDFORD *et al.* 2012; BEDFORD *et al.* 2015). In this situation, we viewed U_b as a free fitting parameter. We adjusted parameters a and U_b to fit our predictions for c and T_{MRCA2} (Fig. 4A). The influenza incidence predicted by our analysis (7%) agrees with the experimental value (PARK *et al.* 2009). Furthermore, our best-fit value a =14.7 is also in excellent agreement with the observed value (PARK *et al.* 2009) used in previous stochastic simulation of a similar model by (BEDFORD *et al.* 2012) (in their notation, $1/s$). Further, our best-fit estimate of U_b at 3.3e-4 is 3.3-fold larger than that in the simulation (BEDFORD *et al.* 2012). Thus, our method allows us to obtain a more effective epitope length at the population level, L~7 variable aminoacids.

Thus, our analytic method enables accurate estimates of four important population-scale parameters: a, L, U_b, and the annual incidence of influenza A H3N2 virus infection by combining epidemiological and genomic data. Three of these parameters (a, L and the incidence) are known from data analysis and agree with with previous data.

Our analysis is not restricted to influenza. We also calculated the predicted three parameters (c, T_{MRCA2}, and the incidence) over a range of values of N and a (Fig 4B, main text), as well the sensitivity of the speed and the incidence to parameters U_b and R_0 (Figs. S3 and S4).

Conclusion

We show that the effective selection coefficient σ and the substitution rate c are approximately proportional to R_0/a (Eqs. 10-14, main text). We observe a logarithmic dependence of population size N and mutation rate U_b for all these parameters, a watermark of asexual evolution models (ROUZINE *et al.* 2001; ROUZINE *et al.* 2003; DESAI AND FISHER 2007; DESAI *et al.* 2007; GOOD *et al.* 2012; ROUZINE AND WEINBERGER 2013; ROUZINE *et al.* 2014a; ROUZINE *et al.* 2014b)

References

Astier, S., 2007 *Principles of Plant Virology*. Science Publishers.

Batorsky, R., R. A. Sergeev and I. M. Rouzine, 2014 The route of HIV escape from immune response targeting multiple sites is determined by the cost-benefit tradeoff of escape mutations. PLoS Computational Biology 10: e1003878.

Bedford, T., A. Rambaut and M. Pascual, 2012 Canalization of the evolutionary trajectory of the human influenza virus. BMC Biol 10: 38.

Bedford, T., S. Riley, I. G. Barr, S. Broor, M. Chadha *et al.*, 2015 Global circulation patterns of seasonal influenza viruses vary with antigenic drift. Nature 523: 217-220.

Desai, M. M., and D. S. Fisher, 2007 Beneficial mutation selection balance and the effect of linkage on positive selection. Genetics 176: 1759-1798.

Desai, M. M., D. S. Fisher and A. W. Murray, 2007 The speed of evolution and maintenance of variation in asexual populations. Curr Biol 17: 385-394.

Fonville, J. M., S. H. Wilks, S. L. James, A. Fox, M. Ventresca *et al.*, 2014 Antibody landscapes after influenza virus infection or vaccination. Science 346: 996-1000.

Good, B. H., I. M. Rouzine, D. J. Balick, O. Hallatschek and M. M. Desai, 2012 Distribution of fixed beneficial mutations and the rate of adaptation in asexual populations. Proc Natl Acad Sci U S A 109: 4950-4955.

Janeway, C. A., and P. Travers, 1996 *Immunobiology. The immune system in health and disease*. Current Biology Ltd./Garland Publishing Inc., London/New York.

Koel, B. F., D. F. Burke, T. M. Bestebroer, S. van der Vliet, G. C. Zondag *et al.*, 2013 Substitutions near the receptor binding site determine major antigenic change during influenza virus evolution. Science 342: 976-979.

Lassig, M., 2012 Chance and risk in adaptive evolution. Proc Natl Acad Sci U S A 109: 4719-4720.

Lin, J., V. Andreasen, R. Casagrandi and S. A. Levin, 2003 Traveling waves in a model of influenza A drift. Journal of theoretical biology 222: 437-445.

Luksza, M., and M. Lassig, 2014 A predictive fitness model for influenza. Nature 507: 57-61.

Neher, R. A., and O. Hallatschek, 2013 Genealogies of rapidly adapting populations. Proceedings of the National Academy of Sciences of the United States of America 110: 437-442.

Nowak, m. a., 2006 *Evolutionary Dynamics: Exploring the Equations of Life*. Harvard University Press.

Park, A., J. Daly, N. Lewis, D. Smith, J. Wood *et al.*, 2009 Quantifying the impact of immune escape on transmission dynamics of influenza. Science 326: 726-728.

Poulin, R., 2007 *Evolutionary Ecology of Parasites*. Princeton University Press.

Rice, S. H., 2004 *Evolutionary Theory: Mathematical And Conceptual Foundations*. Sinauer Associated.

Rouzine, I. M., E. Brunet and C. O. Wilke, 2008 The traveling-wave approach to asexual evolution: Muller's ratchet and speed of adaptation. Theor Popul Biol 73: 24-46.

Rouzine, I. M., J. M. Coffin and L. S. Weinberger, 2014a Fifteen Years Later: Hard and Soft Selection Sweeps Confirm a Large Population Number for HIV In Vivo. PLoS Genetics 10 e1004179.

Rouzine, I. M., B. S. Razooky and L. S. Weinberger, 2014b Stochastic variability in HIV affects viral eradication. Proc Natl Acad Sci U S A 111: 13251-13252.

Rouzine, I. M., A. Rodrigo and J. M. Coffin, 2001 Transition between stochastic evolution and deterministic evolution in the presence of selection: general theory and application to virology [review]. Microbiol. Mol. Biol. Rev. 65: 151-185.

Rouzine, I. M., J. Wakeley and J. M. Coffin, 2003 The solitary wave of asexual evolution. Proc Natl Acad Sci U S A 100: 587-592.

Rouzine, I. M., and L. Weinberger, 2013 The quantitative theory of within-host viral evolution [review]. J. Stat. Mech.: Theory and Experiment: P01009.

Smith, D., A. Lapedes, J. de Jong, T. Bestebroer, G. Rimmelzwaan *et al.*, 2004 Mapping the antigenic and genetic evolution of influenza virus. Science 305: 371-376.

Tsimring, L. S., H. Levine and D. Kessler, 1996 RNA virus evolution via a fitness-space model. Phys. Rev. Lett. 76: 4440-4443.

Walczak, A. M., L. E. Nicolaisen, J. B. Plotkin and M. M. Desai, 2012 The structure of genealogies in the presence of purifying selection: a fitness-class coalescent. Genetics 190: 753-779.
