# Supplementary figures and images for: Antigenic evolution of viruses in host populations
Source: PLoS Pathog. 2018 Sep 12;14(9):e1007291. doi: 10.1371/journal.ppat.1007291 (PMC6173453; doi:10.1371/journal.ppat.1007291)

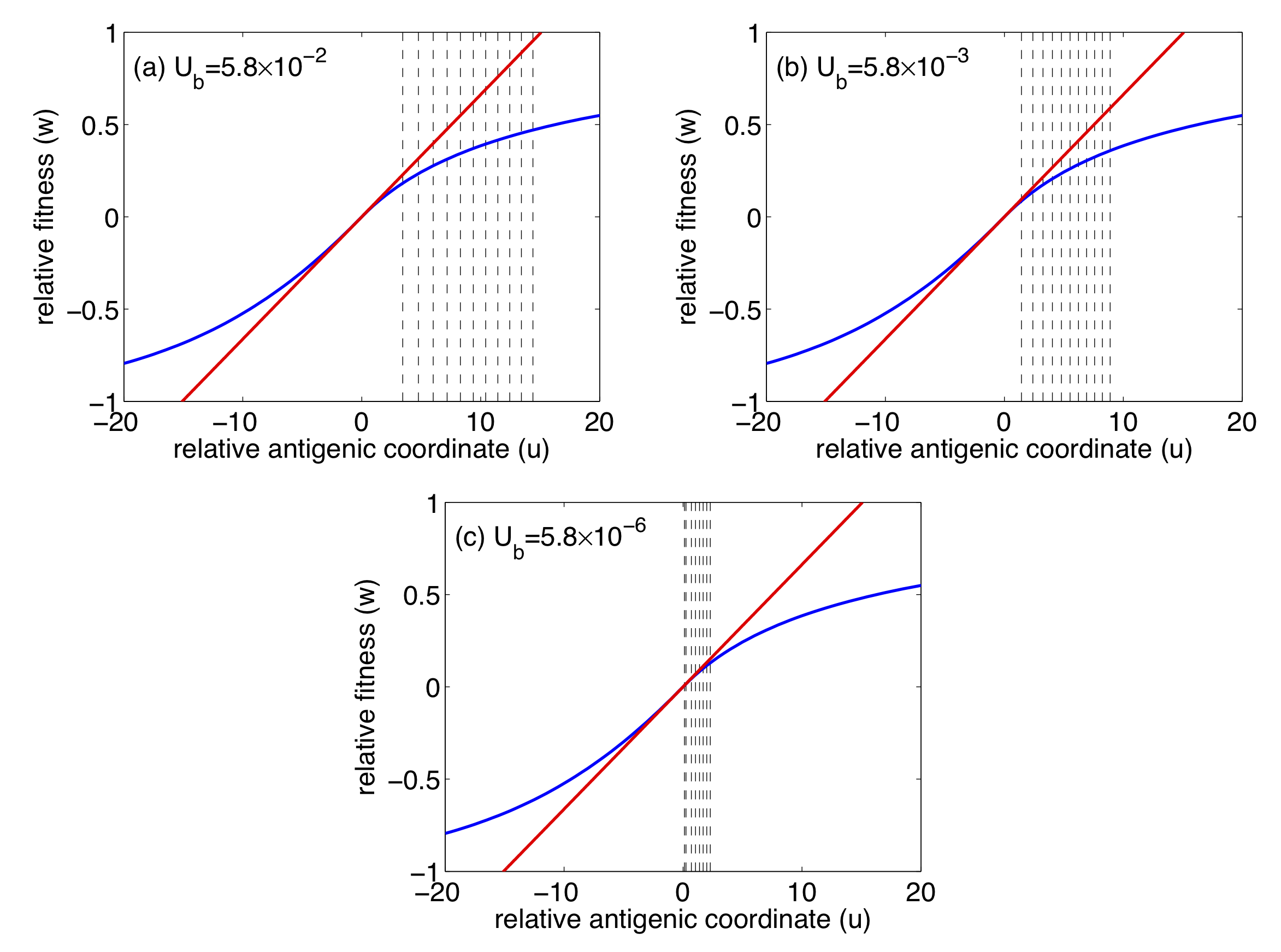

Supplement: S1 Fig — (TIFF) [file ppat.1007291.s002.tiff]

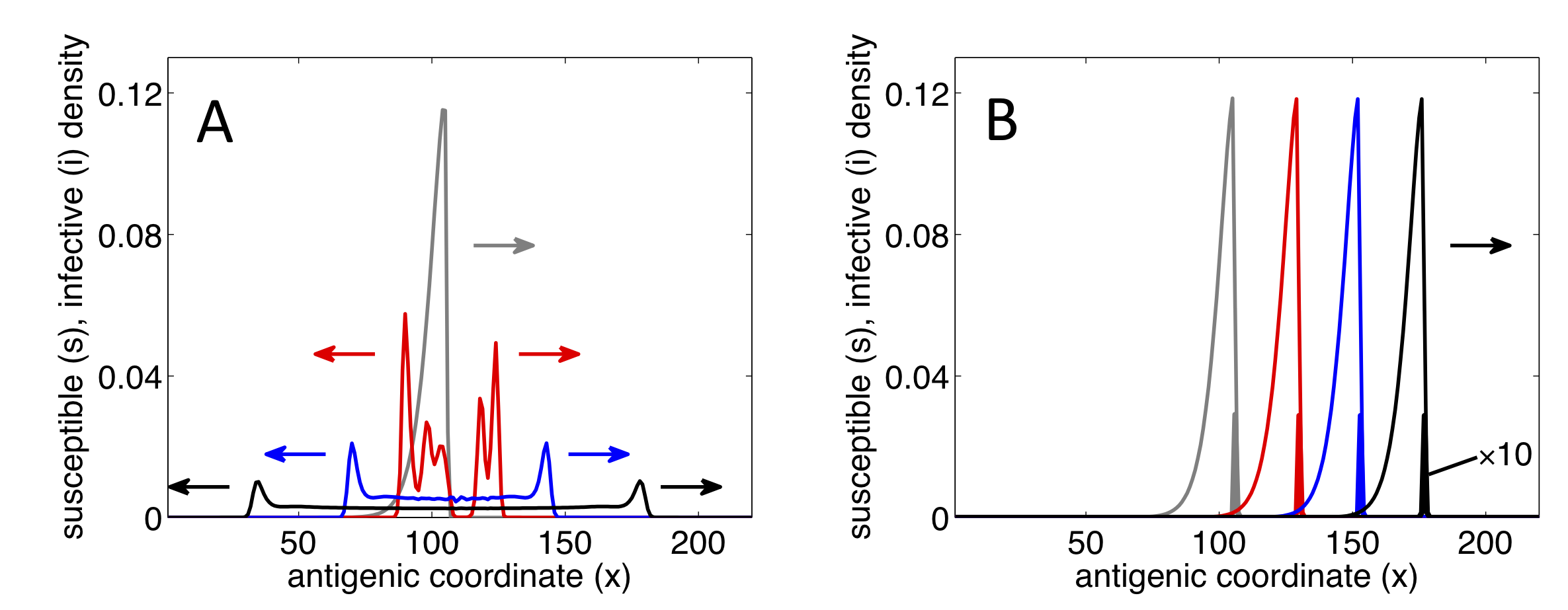

Supplement: S2 Fig — (TIFF) [file ppat.1007291.s003.tiff]

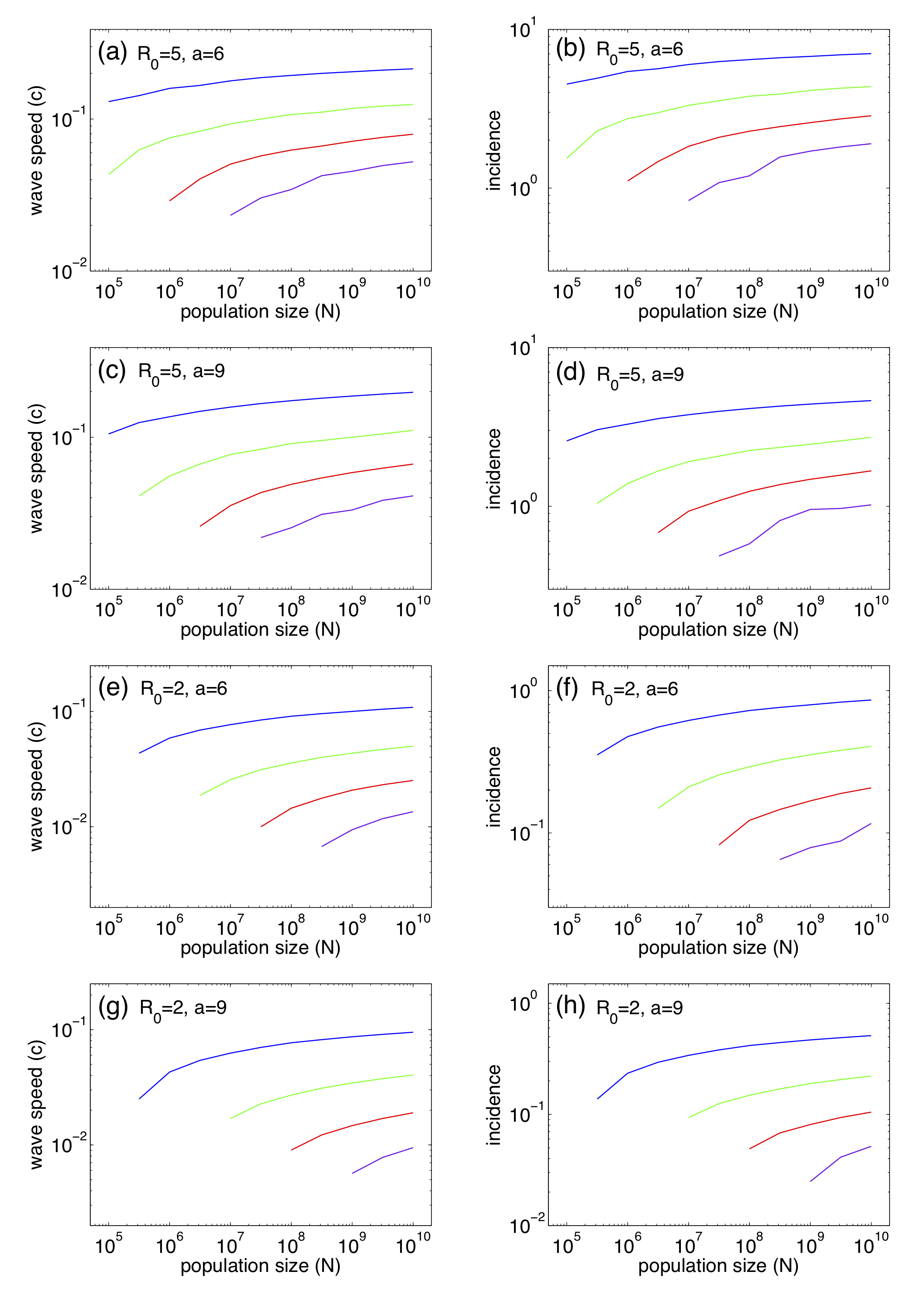

Supplement: S3 Fig — (TIFF) [file ppat.1007291.s004.tiff]

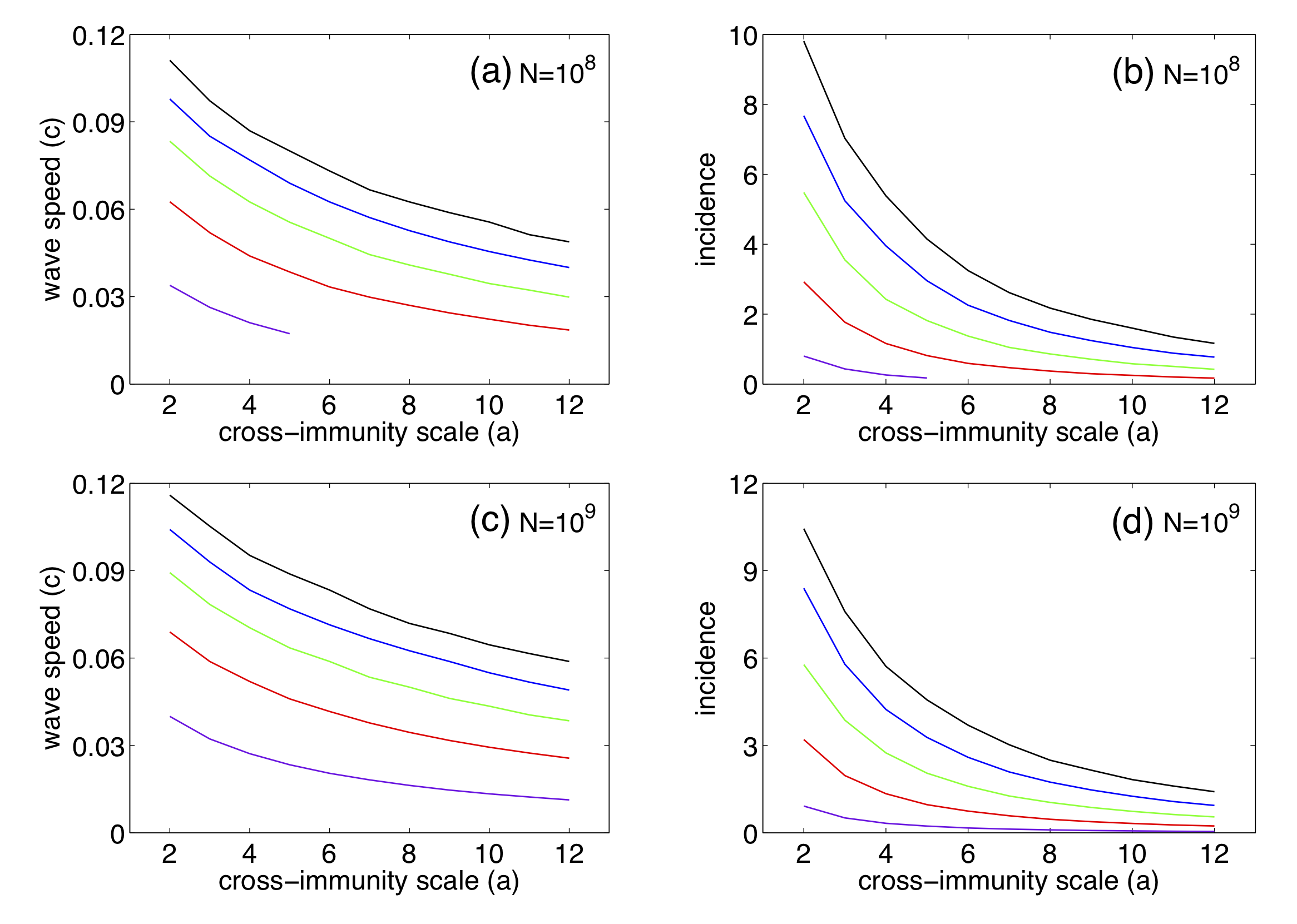

Supplement: S4 Fig — (TIFF) [file ppat.1007291.s005.tiff]

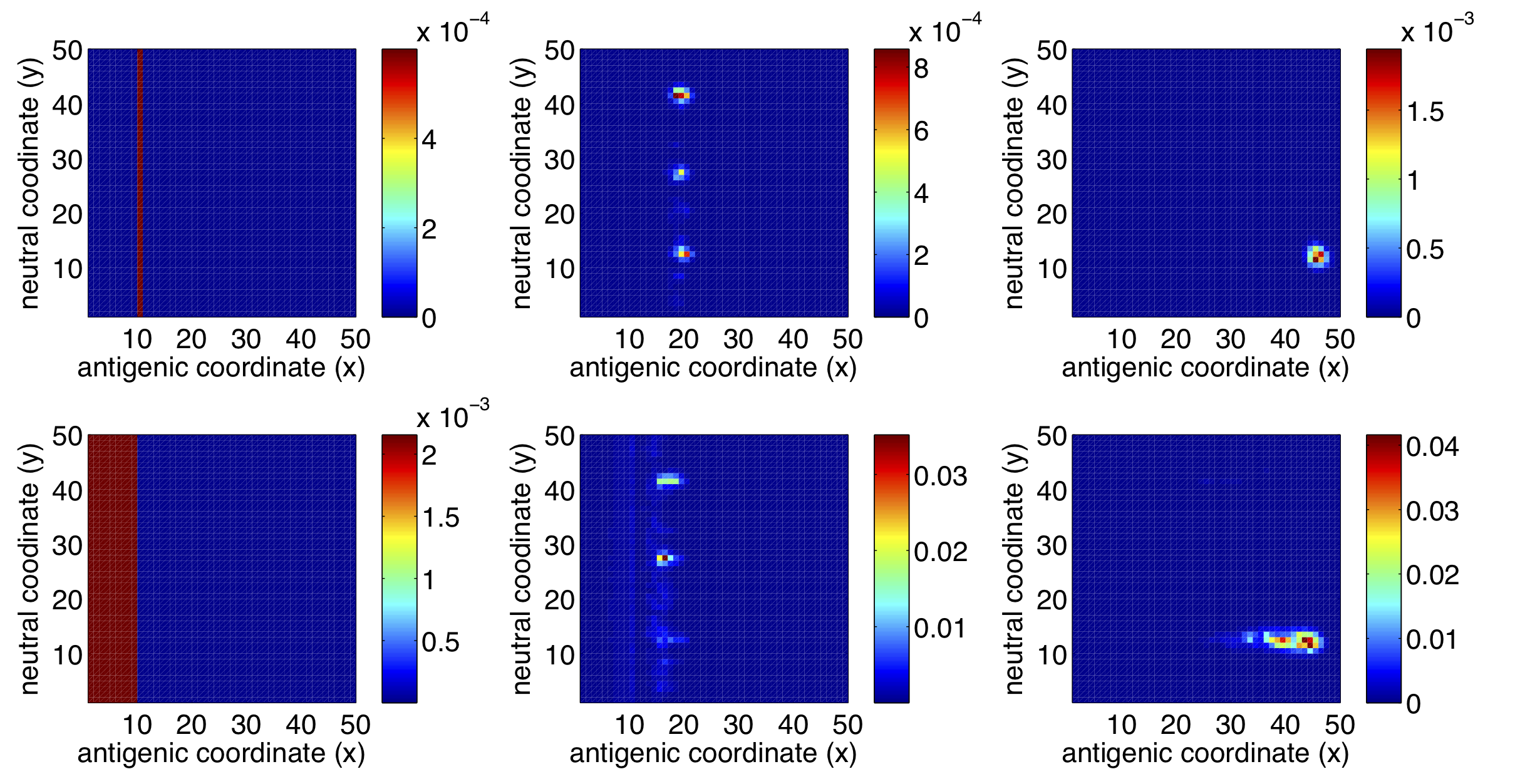

Supplement: S5 Fig — (TIFF) [file ppat.1007291.s006.tiff]

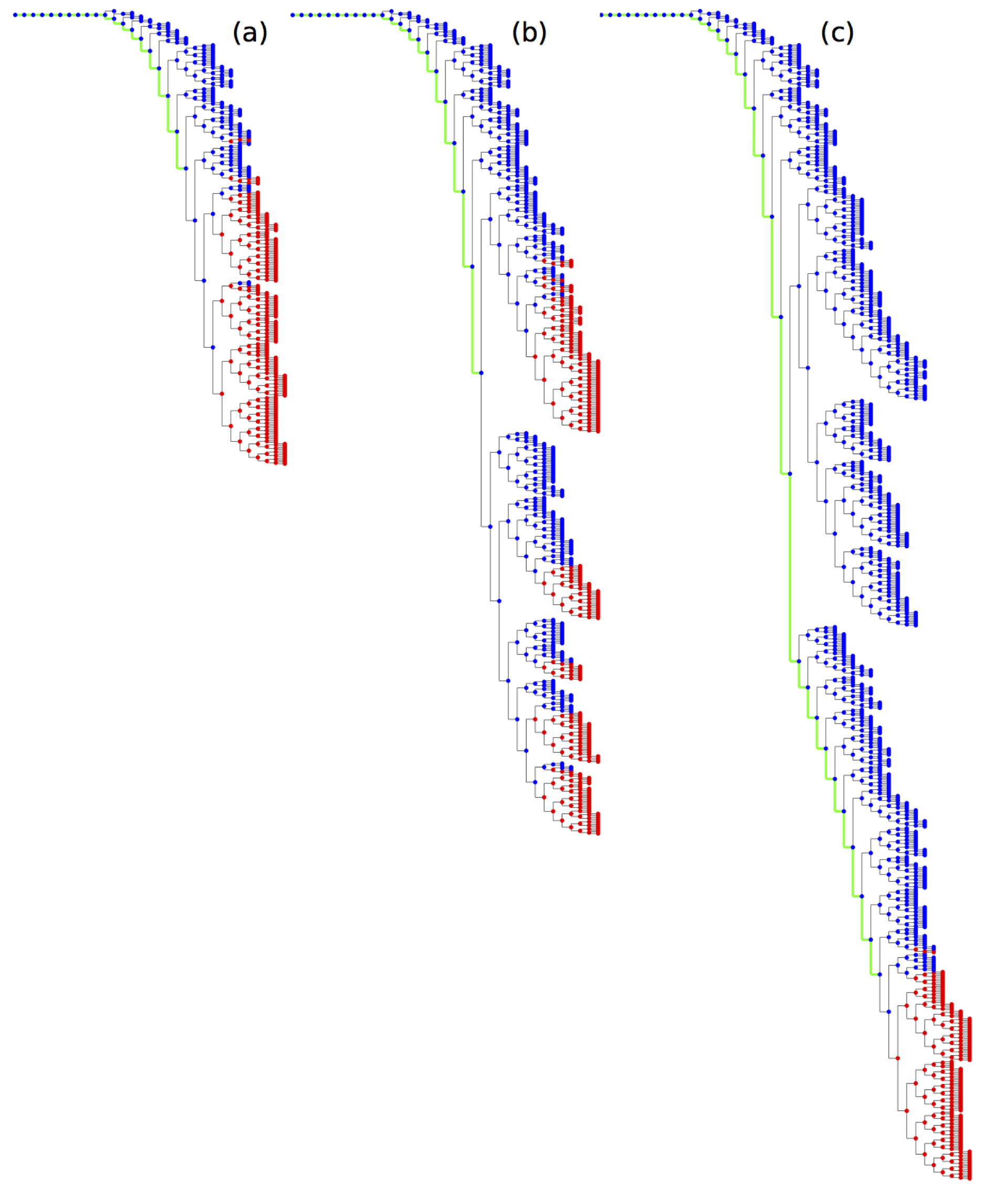

Supplement: S6 Fig — (TIFF) [file ppat.1007291.s007.tiff]
